# Systematic review of the effects of care provided with and without diagnostic clinical prediction rules

**DOI:** 10.1186/s41512-017-0013-2

**Published:** 2017-04-26

**Authors:** Sharon L. Sanders, John Rathbone, Katy J. L. Bell, Paul P. Glasziou, Jenny A. Doust

**Affiliations:** 1 Centre for Research in Evidence-Based Practice, Bond University, Gold Coast, Queensland Australia; 2 Sydney School of Public Health, Sydney Medical School, The University of Sydney, Sydney, Australia

**Keywords:** Clinical prediction rules, Impact analysis, Systematic review

## Abstract

**Background:**

Diagnostic clinical prediction rules (CPRs) are worthwhile if they improve patient outcomes or provide benefits such as reduced resource use, without harming patients. We conducted a systematic review to assess the effects of diagnostic CPRs on patient and process of care outcomes.

**Methods:**

We searched electronic databases and a trial registry and performed citation and reference checks, for randomised trials comparing a diagnostic strategy with and without a CPR. Included studies were assessed for risk of bias and similar studies meta-analysed.

**Results:**

Twenty-seven studies evaluating diagnostic CPRs for 14 conditions were included. A clinical management decision was the primary outcome in the majority of studies. Most studies were judged to be at high or uncertain risk of bias on ≥3 of 6 domains. Details of study interventions and implementation were infrequently reported.

For suspected Group A *Streptococcus* throat infection, diagnostic CPRs reduced symptoms (1 study) and antibiotic prescriptions (5 studies, RR 0.86, 95% CI 0.75 to 0.99). For suspected cardiac chest pain, diagnostic strategies incorporating a CPR improved early discharge rates (1 study), decreased objective cardiac testing (1 study) and decreased hospitalisations (1 study). For ankle injuries, Ottawa Ankle Rules reduced radiography when used with clinical examination (1 study) but had no effect on length of stay as a triage test (1 study). For suspected acute appendicitis, CPRs had no effect on rates of perforated appendix (1 study) or the number of non-therapeutic operations (5 studies, RR 0.68, 95% CI 0.43 to 1.08). For suspected pneumonia, CPRs reduced antibiotic prescribing without unfavourable outcomes (3 studies). For children with possible serious bacterial infection, diagnostic CPRs did not improve process of care outcomes (3 studies).

**Conclusion:**

There are few randomised trials of diagnostic CPRs, and patient outcomes are infrequently reported. Diagnostic CPRs had a positive effect on process outcomes in some clinical conditions; however, many studies were at unclear or high risk of bias and the results may be context specific. Future studies should seek to detail how the CPR might alter the diagnostic pathway, report effects on both patient and process outcomes, and improve reporting of the study interventions and implementation.

**Trial registration:**

The protocol for this review was not registered with PROSPERO, the international prospective register of systematic review protocols. The review was conceived and protocol prepared prior to the launch of PROSPERO in February 2011.

**Electronic supplementary material:**

The online version of this article (doi:10.1186/s41512-017-0013-2) contains supplementary material, which is available to authorized users.

## Background

Diagnostic clinical prediction rules (CPRs) are tools intended to supplement clinicians’ diagnostic reasoning and judgment [1] by providing an estimate of the probability of the presence of a particular disease in an individual and/or by suggesting a course of clinical action based on the underlying probability estimate.

The decision to introduce a diagnostic CPR into practice should ideally be based on evidence that implementation leads to either (1) improved patient outcomes or (2) other benefits such as reduced resource use, relative to the current alternative pathway, without adversely affecting patients. The vast majority of studies of diagnostic CPRs in the literature, however, have focused on establishing the accuracy of the CPR relative to a reference standard test in derivation and validation studies, with no comparison to the existing diagnostic pathway. Often this information is used to decide on the clinical usefulness of the CPR. However, diagnostic accuracy does not necessarily translate into patient benefits [2], nor is it a necessary prerequisite for improved patient health as a CPR may alter patient outcomes through other non-decisional routes including by changing the timing of decisions and actions relative to the existing pathway or through direct effects of the CPR itself [3, 4]. Therefore, impact studies of diagnostic CPRs are necessary. These studies compare testing strategies with and without a diagnostic CPR reporting relevant patient and/or process of care outcomes [5].

Whether implementation of current, validated diagnostic CPRs leads to more benefit than harm is unclear. The effects of CPRs as part of a broader group of clinical decision support tools (computerised and non-computerised tools for improving clinical decision-making including, among other things, prediction rules, guideline-based recommendations, alerts or reminders, condition-specific order sets and contextually relevant reference information) have been extensively reviewed [6–10]. However, the effect of prediction rules specifically is difficult to discern from these reviews as the effects have not been analysed according to the type of clinical decision support system implemented. A recent systematic review provides some insight into the impact of both prognostic and diagnostic CPRs relevant to primary care [11]. This review included randomised and non-randomised studies evaluating CPRs as a stand-alone intervention identified from a register of CPRs considered relevant to primary care [12]. This review found that relatively few CPRs for primary care had gone through impact analysis and that a patient outcome was rarely the primary outcome. To our knowledge, there has been no review of the effect of diagnostic CPRs alone that have been developed for a range of conditions commonly encountered in clinical medicine and used as a stand-alone tool or as part of a care pathway, and including only studies of randomised design. Such a review may inform the selection and implementation of diagnostic CPRs in a wide range of practice settings and guide future diagnostic CPR research.

To determine the effect of diagnostic CPRs on patient and process outcomes, we reviewed studies randomly allocating clinicians or patients to care provided with a diagnostic CPR or to care without a diagnostic CPR.

## Methods

We conducted the systematic review according to the PRISMA statement reporting guidelines (see Additional file 1).

### Data sources and searches

We searched MEDLINE and The Cochrane Central Register of Controlled Trials (CENTRAL) to September 2016 using MeSH and text word terms for the intervention and a study design filter (Additional file 2). We checked systematic reviews of diagnostic clinical prediction rules and clinical decision support systems identified through searching PubMed Clinical Queries. Reference lists of studies obtained in full text were checked, and studies included in the review were forward searched using the Science Citation Index Expanded in Web of Science. The International Clinical Trials Registry Platform (ICTRP) was searched (September 2016) to identify trials planned, in progress or recently completed.

### Study inclusion and exclusion criteria

We included randomised controlled trials allocating clusters of individuals, or individual clinicians or patients, to a group ‘exposed’ to a diagnostic strategy comprised of or incorporating a previously derived diagnostic CPR (experimental) or to care provided without a CPR (control) that measured the impact of the CPR on patient outcomes and/or health care processes.

Eligible experimental interventions comprised the provision of a diagnostic CPR or the output of it or a diagnostic strategy incorporating a diagnostic CPR (for example a strategy including a CPR and another laboratory or imaging test) to a clinician. A diagnostic CPR was defined as a combination of variables obtained from history, examination or diagnostic testing, developed using a statistical method and which provides a probability of the presence of disease for an individual and/or suggested a diagnostic or therapeutic course of action based on the underlying probability, such as further testing or management or both. We excluded studies evaluating tools incorporating a CPR designed for use by the patient or as part of joint decision-making by the clinician and patient. The control intervention was an alternative diagnostic test or testing pathway that did not incorporate the diagnostic CPR. Studies reporting diagnostic accuracy as the primary outcome were included if a current and adequate reference standard was used.

Titles and abstracts were screened by one reviewer and obviously irrelevant articles excluded. A second reviewer independently screened 15% of the titles and abstracts. The second reviewer did not identify any titles or abstracts as potential inclusions that were ultimately included in the review, but considered not relevant by the first reviewer. Full-text articles of potentially relevant studies were obtained and independently assessed by two reviewers against the review inclusion criteria. Discrepancies were resolved through discussion with a third reviewer.

### Data extraction

Two reviewers independently extracted data from the included studies. We extracted information on the experimental arm including:

- The prediction rule or diagnostic strategy tested and its role in the existing diagnostic pathway (replacement, triage or add-on) [13]
- Whether the strategy was assistive (e.g. provided a probability estimate or risk classification) or directive (e.g. suggested or recommended a course of action)
- Whether the use of the prediction tool was discretionary (e.g. the study methods stated the clinician could choose to use or not use the prediction tool) or expected (e.g. the study methods implied or stated that the prediction tools were to be used by clinicians)
- Whether application of the output of the CPR (when the CPR output was a suggested course of action) was discretionary (e.g. the study report stated a clinician could decide whether to follow the rule recommendation or override it) or mandatory (e.g. the recommendation of the CPR was followed in all patients)

For the control arm of the studies, we extracted the description of care as reported by the study authors. For studies describing the control arm only as ‘usual practice’ or similar, we noted whether the study design may have led to some modification of usual practice (for example where clinicians in the control group may have received training or information on the CPRs under evaluation).

We also assessed whether elements of the study interventions necessary for interpretation of study findings and replication in clinical practice were reported. We determined the minimum items required for reporting of the interventions through discussion and consideration of internationally accepted standards for reporting of clinical trials [14, 15]. This included a description of the diagnostic strategies tested (beyond stating the name of the test), a description of the criteria used for establishing a diagnosis or treatment decision and, for studies reporting primary outcomes affected by administration of selected treatments (e.g. patient symptoms), a description of the administered treatment. For the experimental arm, reporting of aspects of implementation (e.g. training in or exposure to the diagnostic strategy) was also assessed. The items were judged as ‘reported’ if any relevant information was described.

### Risk of bias assessment

Two reviewers independently assessed risk of bias according to the Cochrane Risk of Bias Tool [16]. This domain-based evaluation involved independent assessment of risk of bias due to selection, performance, detection, attrition, reporting and others. We considered the following features to judge the risk of bias for each domain: random sequence generation, allocation concealment (selection bias), blinding of participants (performance bias), blinding of outcome assessors (detection bias), incomplete outcome data (attrition bias) and selective reporting (reporting bias). We assessed the methods as low risk, high risk or unclear risk for each domain. We resolved any disagreement through discussion with a third reviewer. Assessments of risk of bias arising from allocation concealment were based on the methods used to assign clusters of individuals (hospitals or practices), individual clinicians or individual patients to experimental or control groups. Judgments on the likelihood of detection bias were based on details about how outcomes were determined, ascertained or verified and the subjectivity of the primary outcome of the study. For trials that randomised centres, and trials that randomised individual clinicians who then recruited participants to the study, we also assessed risk of bias arising from the recruitment of patients to the study by clinicians aware of their allocation (recruitment bias). For these studies, we also assessed whether the analysis had been adjusted for clustering and whether there was baseline comparability of clusters or statistical adjustment where there was imbalance. The potential for contamination was also recorded. Contamination may occur, for instance, when patients are randomised to either the intervention or control group with the clinician switching between use and no use of the prediction rule, or when clinicians within the same centre randomised to different study groups discuss their experiences. Assessments of risk of bias arising from incomplete outcome data were based on both the number of missing outcomes and the reasons for the missing outcomes with studies considered to be at low risk of attrition bias if there was no missing outcome data, or if the missing outcome data was balanced between study arms and the reasons given for the missing data did not have different implications for each study arm. Selective reporting within each study was assessed by comparing outcomes listed in the study protocol and the outcomes presented in the published report. When a study protocol was not available, the outcomes described in the method section of the published report were compared to the outcomes presented in the results section.

### Data synthesis and presentation and exploration of heterogeneity

Given differences in the objectives of the included trials and CPRs applicable to each condition, we expected heterogeneity in the outcomes reported between and within clinical conditions. To facilitate interpretation, we grouped the included studies by clinical condition. We extracted data on the following types of outcomes: (a) patient outcomes, such as mortality, clinical events, health-related quality of life, patient symptoms or adverse events; (b) health care service outcomes, such as length of stay or time to operation; (c) effect on clinical decisions (test ordering, treatment or referral decisions); (d) appropriateness of clinical decisions (e.g. discharge from care and no serious adverse events); (e) diagnostic accuracy (agreement with a reference standard test) and (f) adherence and implementation outcomes (e.g. use of the tool or compliance with the output of a directive CPR).

For dichotomous outcomes, we presented the adjusted estimates of effect reported in the paper and calculated risk ratios and 95% confidence intervals when this was possible and not presented. For instances where the intervention is intended to prevent an undesirable outcome (e.g. symptoms or antibiotic prescribing), an OR, HR or RR of <1 indicates the intervention is better than the control. Where the intervention is intended to promote a positive event (e.g. safe discharge), an OR, RR or HR >1 confirms treatment efficacy. Continuous outcome measures are presented as reported in the paper. For each study, we identified the primary outcome. The primary outcome was considered to be either the outcome stated by the study as being the primary outcome or the outcome for which a power calculation was conducted. In the absence of these, the primary outcome was considered to be the outcome mentioned in the study objective or reported first in the results section. Statistical analysis was carried out using the Cochrane Collaboration’s Review Manager (Version 5.3) software. Based on difficulties arising from meta-analysing a small number of studies, we combined results when five or more studies for a clinical condition assessed the same outcome, in the same manner, regardless of the specific prediction tool or diagnostic strategy, to obtain a pooled estimate of effect using the general inverse variance method [17]. We chose the risk ratio, a relative measure of effect, as the summary statistic for its ease of interpretation [16]. We considered clinical heterogeneity sufficient to expect that the underlying treatment effects differed between trials. Consequently, we used a random effects model to produce an overall summary of the average treatment effect across the included studies. For studies including two experimental arms (e.g. CPR and CPR plus rapid antigen detection test (RADT)) [18, 19], we included data only from the CPR-alone arm in the meta-analysis. For the one study including two control arms (e.g. clinical judgment with no diagnostic aid or with a standardised data collection form) [20], we included data only from the clinical judgment with no diagnostic aid arm. To pool individual and cluster randomised trials in the same model, adjustment for clustering was conducted. Adjustment involved reducing the size of the cluster trials to the effective sample size by dividing the sample size by the design effect, where the design effect is equal to 1 + (*m* − 1) × intracluster correlation coefficient (ICC) and *m* is the average cluster size [16]. To calculate the design effect for studies of sore throat, we used the intracluster correlation coefficient reported in a duplicate publication of one of the other sore throat studies [21]. For appendicitis studies, we used the median ICC reported for implementation research studies reporting process variables (ICC 0.063) and undertook sensitivity analysis using the extremes of the interquartile range [22].

## Results

### Study selection

**Fig. 1:**
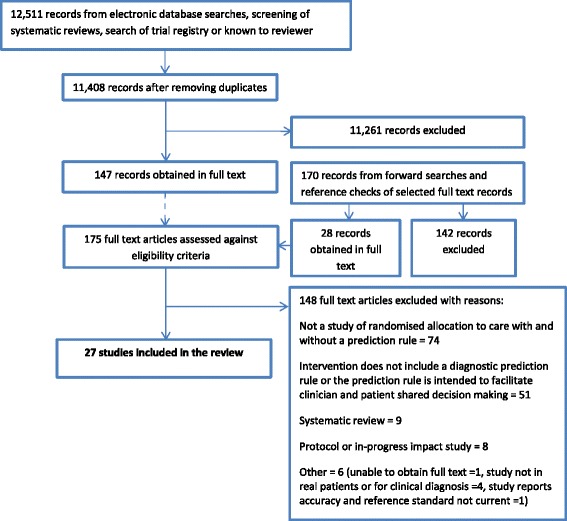
Study flow diagram

Of 12,511 titles and abstracts screened, 175 were obtained in full text and 27 studies were included in the review (Fig. 1) [18–20, 23–46]. The 148 full-text excluded studies are presented in Additional file 3 with reasons for their exclusion.

### Study characteristics

Characteristics of the 27 included studies, grouped by the clinical condition for which the CPR was developed, are presented in Table 1.

**Table 1:** Characteristics of the included studies by clinical condition

Study/location/setting	Diagnostic strategies tested		Proposed role of the CPR or experimental diagnostic strategy	Use of the CPR or experimental diagnostic strategy	Application of the output of the CPR or experimental diagnostic strategy	Primary outcome of the study^a^
	Study arm	Interventions (output format of the CPR or diagnostic strategy)				
Group A *Streptococcus* infection of the throat						
Worrall et al. 2007 [19]/Canada/PC	Experimental	Clinicians’ usual practice + Centor Score (D)	Add-on	Expected	Discretionary	Clinical decision
	Experimental	Clinicians’ usual practice + Centor Score + RADT (D)				
	Control	Clinicians’ usual practice				
McIsaac and Goel 1998 [33]/Canada/PC	Experimental	Clinician + Centor Score (D)	Replacement	Expected	Discretionary	Clinical decision
	Control	Clinician + structured clinical checklist				
McIsaac et al. 2002 [34]/Canada/PC	Experimental	Clinician + modified Centor Score (D)	Replacement	Expected	Discretionary	Clinical decision
	Control	Clinician + structured clinical checklist				
McGinn et al. 2013 [32]/USA/PC^b^	Experimental	Clinicians’ usual care + Walsh Rule (D)	Add-on	Discretionary	Discretionary	Clinical decision
	Control	Clinicians’ usual care^c^				
Little et al. 2013 [18]/UK/PC	Experimental	Clinician + FeverPAIN score (D)	Replacement	Expected	Discretionary	Patient outcome
	Experimental	Clinician + FeverPAIN score + RADT (D)				
	Control	Clinician + strategy of delayed antibiotics				
Acute appendicitis						
Douglas et al. 2000 [25]/Australia/SU	Experimental	Clinicians’ clinical diagnosis + Alvarado score + US (D)	Add-on	Expected	Discretionary^d^	Process of care
	Control	Clinicians’ clinical diagnosis^c^				
Farahnak et al. 2007 [27]/Iran/ED	Experimental	Clinicians’ assessment + Alvarado score (D)	Add-on	Expected	Discretionary	Process of care
	Control	Clinicians’ assessment				
Lintula et al. 2010 [31]/Finland/ED	Experimental	Clinicians’ assessment + Lintula score (D)	Add-on	Expected	Discretionary	Accuracy
	Control	Clinicians’ assessment^c^				
Lintula et al. 2009 [30]/Finland/ED	Experimental	Clinicians’ assessment + Lintula score (D)	Add-on	Expected	Discretionary	Accuracy
	Control	Clinicians’ assessment^c^				
Wellwood et al. 1992 [20]/UK/ED	Experimental	Clinicians’ assessment + Leeds decision support system (A)	Add-on/replacement	Expected	NA	Accuracy
	Control	Clinician with no diagnostic aid				
	Control	Clinician + structured data collection form				
Serious bacterial infection in children with fever						
Roukema et al. 2008 [36]/The Netherlands/ED	Experimental	Clinicians’ assessment + prediction rules of Bleeker^e^ (D)	Add-on	Expected	Discretionary	Process of care
	Control	Clinicians’ assessment				
Lacroix et al. 2014 [44]/Switzerland/ED	Experimental	Clinician + LAB score (procalcitonin, CRP, urinary dipstick) (D) blind to WBC count and differential	Replacement	Expected	Discretionary	Clinical decision
	Control	Clinician + WBC count, band count and CRP, blind to procalcitonin and LAB score				
de Vos-Kerkhof et al. 2015 [43]/The Netherlands/ED	Experimental	Clinicians’ usual care + Rule of Nijman (D)	Add-on	Expected	Discretionary	Clinical decision
	Control	Clinicians’ usual care				
Acute coronary syndrome						
Than et al. 2014 [40]/New Zealand/ED	Experimental	Accelerated diagnostic pathway: TIMI score, ECG + troponin at presentation and 2 h after symptom onset (D)	Replacement	Expected	Discretionary	Clinical decision
	Control	Standard-care chest pain pathway: initial ECG + troponin at presentation and 6–12 h after symptom onset				
Sanchis et al. 2010 [37]/Spain/ED	Experimental	Sanchis risk score + NT-proBNP (D)	Replacement	Expected	Discretionary	Process of care
	Control	Chest pain unit protocol with early exercise testing				
Mahler et al. 2015 [46]/USA/ED	Experimental	HEART Pathway: HEART score (including ECG) + troponin at presentation and 3 h later (D)	Replacement	Expected	Discretionary	Clinical decision
	Control	Clinicians’ encouraged to follow current guidelines				
Bacterial pneumonia						
Ferrero et al. 2015 [45]/Argentina/OC	Experimental	Bacterial pneumonia score (D)	Replacement	Expected	Mandatory	Clinical decision
	Control	Standard management based on institutional guidelines				
Torres et al. 2014 [41]/Argentina/OC	Experimental	Bacterial pneumonia score (D)	Replacement	Expected	Mandatory	Clinical decision
	Control	Standard management based on institutional guidelines				
McGinn et al. 2013 [32]/USA/ED^b^	Experimental	Clinicians’ usual care + Walsh Rule (D)	Add-on	Discretionary	Discretionary	Clinical decision
	Control	Clinicians’ usual care^c^				
Ankle/foot fracture						
Auleley et al. 1997 [23]/France/ED	Experimental	Clinicians’ usual practice + Ottawa Ankle Rules (D)	Add-on	Discretionary	Discretionary	Clinical decision
	Control	Clinicians’ usual practice^c^				
Fan et al. 2006 [26]/Canada/ED	Experimental	Ottawa Ankle Rules (D): if positive x-ray, if negative clinical assessment	Triage	Expected	Mandatory	Process of care
	Control	Standard departmental care				
Joint or bone injuries of the extremities in children						
Klassen et al. 1993 [29]/Canada/ED	Experimental	Brand protocol (D): if positive x-ray, if negative clinical assessment	Triage	Expected	Mandatory	Clinical decision
	Control	Standard care				
Suspicious pigmented skin lesion						
Walter et al. 2012 [42]/UK/PC	Experimental	Best practice: history, naked eye examination, seven-point checklist + primary care scoring algorithm + SIAscopy scanner (A)	Add-on	Expected	Discretionary	Clinical decision
	Control	Best practice: history, naked eye examination, seven-point checklist				
Pulmonary embolism						
Rodger et al. 2006 [35]/Canada/NMD	Experimental	Bedside tests (D): Wells’ PE score, D-dimer, AVDSf—if ≥2 tests positive VQ scan	Triage	Expected	Mandatory	Patient outcome
	Control	Initial VQ scan blind to bedside tests				
Gastro-oesophageal reflux disease						
Horowitz et al. 2007 [28]/Israel/PC	Experimental	Algorithm (D): alarm symptom assessment—if positive gastroscopy conducted, if negative GERD score used. If GERD score positive treat, if negative C-urea breath test	Replacement	Expected	Mandatory	Patient outcome
	Control	Doctors’ discretion				
Acute small bowel obstruction						
Bogusevicius et al. 2002 [24]/Lithuania/SU	Experimental	Rule of Bogusevicius (D)	Replacement	Expected	Mandatory	Accuracy
	Control	Contrast radiography				
Clinically important brain injury						
Stiell et al. 2010 [39]/Canada/ED	Experimental	Clinicians’ usual practice + Canadian CT Head Rule (D)	Add-on	Expected	Discretionary	Clinical decision
	Control	Clinicians’ usual practice				
Cervical spine fracture						
Stiell et al. 2009 [38]/Canada/ED	Experimental	Clinicians’ usual practice + Canadian C-Spine Rule (D)	Add-on	Expected	Discretionary	Clinical decision
	Control	Clinicians’ usual practice				

*CPR* clinical prediction rule, *(D)* directive output format, i.e. suggests a course of action, *(A)* assistive output format, i.e. provides a probability without suggesting a course of action, *RADT* rapid antigen detection test, *US* ultrasound, *CRP* C-reactive protein, *AVDSf* alveolar dead-space fraction, *VQ* ventilation-perfusion scan, *PC* primary care, *SU* surgical unit, *ED* emergency department, *OC* outpatient clinic, *NMD* nuclear medicine department

^a^The outcome stated by the study as being the primary outcome or the outcome for which a power calculation was conducted. In the absence of these, the primary outcome was considered to be the outcome mentioned in the study objective or reported first in the results section. Patient outcomes are direct measures of patients health, e.g. symptoms, clinical events. Process of care outcomes are measures of the healthcare provided, e.g. length of stay, time to operation

^b^This study evaluated CPRs for two different clinical conditions

^c^The diagnostic strategy may be modified by the provision of information related to the CPRs being tested

^d^Application mandatory only for certain patients

^e^Different rules for self-referred and clinician-referred patients

Eleven of the 27 included studies were considered to have evaluated a diagnostic CPR or strategy designed to replace the existing approach [18, 24, 28, 33, 34, 37, 40, 41, 44–46], 12 assessed the impact of adding a CPR to the usual diagnostic pathway in order to evaluate the benefit of extra information to diagnostic decision-making [19, 23, 25, 27, 30–32, 36, 38, 39, 42, 43], while three studies evaluated CPRs as a triage test. In the triage studies, the CPR was used before the existing test to determine which patients undergo the existing test [26, 29, 35]. One study with three intervention arms evaluated a CPR as both a replacement and add-on test [20].

In 19 of 27 studies, the CPR was introduced as a stand-alone tool [20, 23, 24, 26–34, 36, 38, 39, 41, 43–45]. The diagnostic CPRs and strategies tested were directive in 25 of the 27 studies, making a recommendation regarding treatment or disposition in 10 studies [19, 24, 27, 30, 31, 37, 40, 41, 44, 45], further diagnostic testing in 8 studies [23, 26, 29, 35, 36, 38, 39, 43] and both further testing and treatment in 7 studies [18, 25, 28, 32–34, 46].

In most studies (18/27), the control group intervention was variably described as ‘clinicians’ assessment’ or ‘usual care’ [19, 23, 25–32, 36, 38, 39, 41–43, 45, 46]. This ranged from control groups in which clinicians were explicitly asked not to change their usual practice, and control groups where clinicians received information on the CPRs being tested in the intervention arm, to control groups where clinicians’ actions were expected to be based on local care guidelines. In the remaining studies, the control group intervention was a standard data collection form, a specific care pathway (e.g. a chest pain clinic protocol or delayed antibiotic strategy) or a single diagnostic test.

Patient outcomes were considered the primary outcome in 3 [18, 28, 35] of the 27 studies, and process of care outcomes (e.g. length of stay) were the primary study outcome in 5 [25–27, 36, 37]. A clinical decision was the primary outcome in 12 studies [19, 21, 23, 29, 32, 38, 39, 41, 43–45, 47], and the appropriateness of the decision was the primary outcome in 3 [34, 40, 42]. Accuracy of a diagnosis or decision was the primary outcome in 4 studies [20, 24, 30, 31]. The types of primary and secondary outcomes reported in the included studies are shown in Additional file 4.

### Risk of bias

**Fig. 2:**
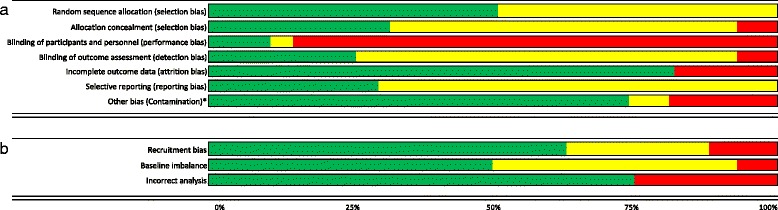
Review authors’ judgments about each risk of bias domain presented as percentages **a** across all included studies and **b** for cluster-randomised studies. Legend:

The majority of studies included in the review (20/27 (74%)) were judged to be at unclear or high risk of bias on 3 or more domains including performance bias. Concealment of allocation, one of the key domains in the assessment of bias, was reported in insufficient detail to enable accurate judgment or was considered inadequate in over half of the included studies (19/27 (70%)). Due to the nature of the intervention, in most studies included in this review, clinicians would have been aware of group allocation. We judged the impact of this on risk of performance bias to be high in the majority (24/27 (89%)) of the included trials and unclear in 1 study. In this study, the interventions were very similar, and the study stated that clinicians were not aware of the alternate interventions. Figure 2 provides a summary of risk of bias judgments for each domain of bias presented as percentages (a) across all included studies and (b) for studies using a cluster randomised design. Details of the risk of bias assessment for each domain of bias for each study are presented in Additional file 5.

### Assessment of the reporting of interventions

We found reporting of interventions in these studies incomplete, with details of the components necessary to replicate the intervention often missing (Additional file 6). When present, they were usually only partly described (for example a diagnostic strategy comprising a CPR and laboratory test described the technique used to perform the laboratory test but did not describe the CPR) or were described with very low level of detail. Only 1 of 25 included studies were judged to have described the diagnostic strategy and criteria for arriving at a diagnosis or decision in both the experimental and control groups [37] and 6 studies to have reported both whether training or exposure to the CPR was provided and the means by which the CPR was implemented into the workflow [23, 26, 32, 36, 42, 43]. Control interventions that were variably described as ‘usual’ care were less frequently described than control interventions comprising more technological procedures (e.g. contrast radiography).

### Effects of diagnostic strategies incorporating diagnostic clinical prediction rules

The estimated effects of exposure to a diagnostic strategy incorporating a diagnostic CPR are presented according to the clinical condition the CPR was developed to detect.

### Studies of diagnostic CPRs for suspected Group A *Streptococcus* throat infection

Tabulated results are presented in Additional file 7.

Five studies evaluated three different CPRs (Walsh, Centor and FeverPAIN score) and a modified version of one (Modified Centor Score) [18, 19, 32–34]. All CPRs evaluated were directive (i.e. provided management recommendations), and in all five studies, application of the output of the CPR or diagnostic strategy was discretionary (i.e. the clinician could follow or not follow the recommendations of the CPR). Four of the five studies were judged to be at high or unclear risk of bias on 3 or more of the 6 key domains.

#### Clinical outcomes

In one study reporting patient-reported symptoms (primary study outcome) and adverse effects [18], there were greater improvements in symptom severity among patients randomised to the FeverPAIN score compared to the control arm with a strategy of delayed antibiotics (mean difference adjusted for baseline symptom severity and fever −0.33 on a score of 0–6, 95% CI −0.64 to −0.02, *p* = 0.04). Symptom resolution was faster among patients randomised to the FeverPAIN score (HR adjusted for baseline symptom severity and fever 1.30, 95% CI 1.03 to 1.63; median duration 5 days (IQR 3–7) in the control arm and 4 days (IQR 2–6) in the score-only arm). There were no differences between the study groups in the proportion returning to the clinic with sore throat within a 1-month period or the occurrence of suppurative complications (none occurred during the study).

#### Clinical decisions

**Fig. 3:**
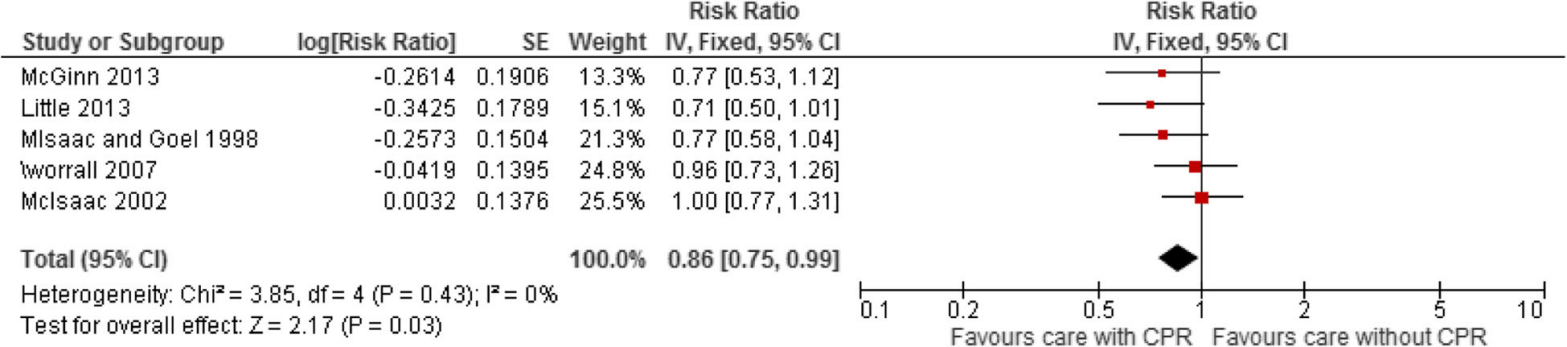
Meta-analysis of Group A *Streptococcus* throat infection studies for the outcome antibiotic prescriptions

All five studies reported clinical decisions to prescribe antibiotics [18, 19, 32–34]. In the three studies reporting clinical decisions to prescribe as the primary study outcome, CPRs were associated with a non-significant reduction in prescribing [19, 32, 33]. One of these studies reported no difference in emergency department or outpatient visits as a proxy for the appropriateness of care [32]. Complications either did not occur or were not reported in the other two studies [19, 33]. In pooled analysis, CPRs reduced antibiotic prescriptions compared to care provided without a CPR (pooled RR 0.86, 95% CI 0.75 to 0.99) (Fig. 3).

The results of studies reporting the effects of diagnostic CPRs on other clinical decisions including test use and the appropriateness of antibiotic prescribing are shown in Additional file 7.

### Studies of diagnostic CPRs for suspected acute appendicitis

Tabulated results are presented in Additional file 7.

Five studies evaluated three different CPRs (Alvarado score, Lintula score and the Leeds decision support system) [20, 25, 27, 30, 31]. The Leeds decision support system was assistive, providing only an estimate of the probability of appendicitis without recommending a course of action, and application of the management recommendations of the Alvarado and Lintula scores was discretionary. All five studies were judged to be at high or unclear risk of bias arising from lack of blinding of care providers and outcome assessors.

#### Clinical outcomes

Perforated appendix rates did not significantly differ between the experimental group and a control group providing care without a diagnostic aid (RR 0.47, 95% CI 0.19 to 1.15) and a control group where clinicians used a standard data collection form (RR 0.81, 95% CI 0.31 to 2.16) [20, 48].

#### Process of care outcomes

The results of two studies providing data on the effect of CPRs on duration of hospitalisation among patients with suspected appendicitis are conflicting. One small study of the Alvarado score reported significantly shorter duration of hospitalisation in the intervention group (median 37.00 vs 60.40 h, *p* = 0.03) ‘without significant increase’ in perforation (one perforation occurred in the intervention group and two perforations in the control group) [27], while the other study reported no difference in mean duration of hospital stay between a diagnostic protocol incorporating the Alvarado score and graded compression ultrasound, and the control group (mean 53.4 vs 54.5 h, *p* = 0.84) [25].

The results of studies reporting the effects of diagnostic CPRs on other process of care outcomes including admission rates and time to surgery are shown in Additional file 7.

#### Clinical decisions/appropriateness of clinical decisions

**Fig. 4:**
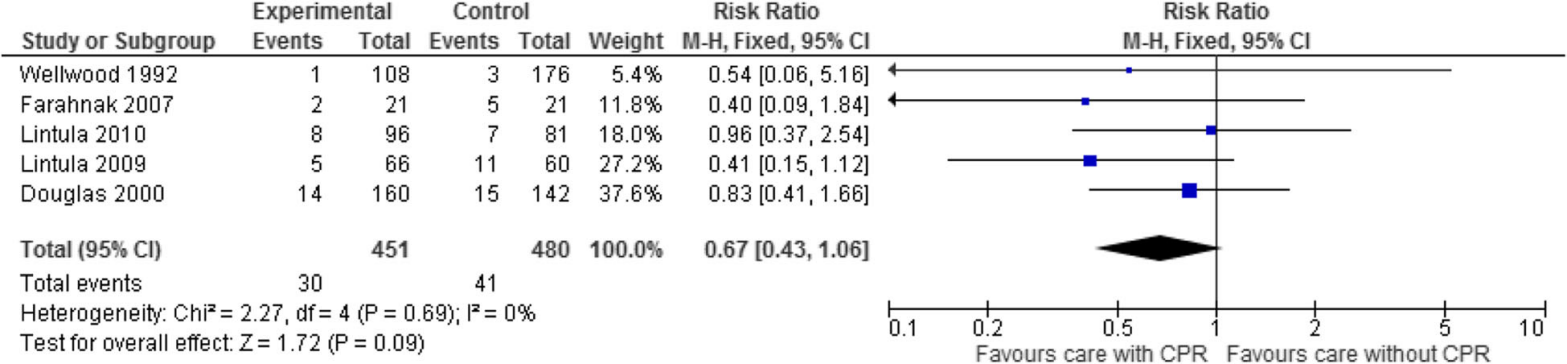
Meta-analysis of appendicitis studies for the outcome unnecessary appendectomies

In pooled analysis of five trials, diagnostic strategies incorporating CPRs reduced unnecessary appendectomy compared to usual clinical assessment, but this was not statistically significant (pooled RR 0.68, 95% CI 0.43 to 1.08) [20, 25, 27, 30, 31]. The direction of effect was consistently in favour of the experimental arms of the trials, though the risk ratios varied widely (Fig. 4). An ICC obtained from an analysis of implementation research studies reporting process outcomes (ICC 0.063) was used to adjust for clustering in the one cluster randomised trial included in this analysis. In sensitivity analysis, using the lower extreme of the ICC interquartile range, in which more weight is given to the study in the meta-analysis similar to that applied when unadjusted data are used, the confidence intervals were narrower and the effect significant (pooled RR 0.64, 95% CI 0.41 to 0.98).

### Studies of diagnostic CPRs for suspected serious bacterial infection in children with fever

Tabulated results are presented in Additional file 7.

Three studies evaluated three different directive CPRs [36, 43, 44]. All three were considered at high risk of bias arising from a lack of blinding of care providers and at unclear risk of bias for allocation concealment. There was a non-statistically significant increased length of stay in the emergency department with diagnostic strategies incorporating a CPR in the two studies reporting this outcome (138 vs 123 median minutes, *p* = 0.16, and 117 vs 114 median minutes, *p* = 0.05 in the experimental and control groups of both studies, respectively) [36, 43]. A CPR comprised of a panel of three laboratory tests (procalcitonin, C-reactive protein and urinary dipstick) did not reduce antibiotic prescribing compared to the judgment of clinicians with access to the results and associated treatment recommendation of the white blood cell count, band count and C-reactive protein tests (prescriptions for 41 and 42% of the experimental and control groups, respectively, *p* = 0.88) [44]. A CPR also had no effect on the use of diagnostic tests when serious bacterial infection was and was not present as judged by a reference standard (60 vs 57% of children without pneumonia in the experimental and control groups, respectively, received chest x-ray, *p* > 0.05, and 67 vs 53% of children without urinary tract infection had urine culture performed, *p* > 0.05) [43]. This CPR resulted in a significant increase in the number of urine dipstick tests, a significant reduction in the number of full blood count tests and a non-significant decrease in the number of diagnostic tests overall compared to the control group.

### Studies of diagnostic CPRs for suspected acute coronary syndromes

Tabulated results are presented in Additional file 7.

Three studies judged to be at low risk of bias evaluated different diagnostic strategies incorporating different CPRs and tests for individuals presenting with possible cardiac chest pain [37, 40, 46]. In the first of these studies, a strategy incorporating a diagnostic CPR (TIMI score), ECG and cardiac troponin testing significantly increased the number of patients successfully discharged within 6 h (discharge was considered successful if it occurred within 6 h of emergency department arrival and patient did not experience a major cardiac adverse event within 30 days) (OR 1.92, 95% CI 1.18 to 3.13) [40], compared with a conventional chest pain protocol. In the second study, a diagnostic strategy comprising a CPR and NT-proBNP test decreased the number of patients hospitalised (OR 0.6, 95% CI 0.4 to 0.9) with no differences in death or myocardial infarction between study groups after 1-year follow-up [37]. In the final study, a strategy incorporating the HEART score with serial troponin measures decreased objective cardiac testing at 30 days (69 vs 57%, *p* = 0.04) compared with usual care [46].

### Studies of diagnostic CPRs for suspected (bacterial) pneumonia

Tabulated results are presented in Additional file 7.

Three studies evaluated different CPRs in patients with suspected pneumonia [32] and in patients (unvaccinated and vaccinated against pneumococcus) with non-severe community-acquired pneumonia (according to clinical criteria) of unknown aetiology [41, 45]. All three studies were judged to be at unclear or high risk of bias on 3 or more domains of bias. Prescriptions for antibiotics were significantly reduced with use of the CPR with no difference in unfavourable outcomes between interventions [32, 41, 45].

### Studies of diagnostic CPRs for suspected ankle or mid-foot fracture

Tabulated results are presented in Additional file 7.

Two studies evaluated the impact of the Ottawa Ankle Rules (OARs) [23, 26]. In one trial, the OARs were used as a triage test [26]. This trial was judged to be at unclear or high risk of bias arising from inadequate randomisation sequence generation, incomplete data and selective reporting. In this trial, standard departmental care was compared to a pathway in which the OARs were applied at presentation: if positive, the patient was x-rayed, and if negative, the patient underwent usual clinical assessment. In the second trial [23], clinicians in hospitals randomised to the intervention were encouraged to use the OARs as part of their clinical assessment. This study was judged to be at high or unclear risk of bias on 5 of the 6 domains of bias assessed and at unclear risk from baseline differences between the randomised groups. When used as a triage test, the OARs did not decrease total length of stay in the emergency department (mean difference −6.7 min, 95% CI −20.9 to 7.4), and there was no difference in patient satisfaction ratings and radiography requests between the study groups [26]. The OARs, when used and applied at the discretion of the clinician as an add-on test, significantly decreased radiography requests (76 vs 99%, *p* = 0.03). Three fractures were later diagnosed among participants who had not received an x-ray—all were randomised to the experimental arm. However, there was no active follow-up in this trial, and participants were only advised to consult again if there was persistent pain or inability to walk so it is possible other fractures may have been missed. In this trial, 96 and 98% of patients in the experimental and control groups, respectively, were satisfied with the care received [23].

### Single studies of diagnostic CPRs for other conditions

Tabulated results are presented in Additional file 7.

#### Clinical outcomes

Two studies of CPRs for different clinical conditions reported clinical outcomes as the primary outcome of the study. In the first, a score-directed treatment algorithm for patients with upper abdominal complaints significantly decreased symptom severity (MD on a scale of 0–10 2.5, 95% CI 1.49 to 3.51) [28]. The other study was an equivalence trial of a triage strategy of ‘bedside tests’ incorporating a diagnostic CPR for directing ventilation-perfusion scanning vs a strategy of scanning all patients. In this study, there was no significant difference in venous thrombotic events among patients not taking anticoagulation agents during follow-up (% difference in venous thromboembolic event rate −0.6, 95% CI −4.1 to 2.9), but the triage strategy excluded pulmonary embolism in 34% of patients (who therefore avoided ventilation-perfusion scanning) and reduced other diagnostic imaging tests performed [35].

#### Clinical decisions/appropriateness of clinical decisions

Radiography requests increased, but time in the emergency department significantly decreased, in a study of a CPR used as a triage test in children with extremity trauma [29]. There was no difference in appropriate referrals in adults with pigmented skin lesions with use of a diagnostic protocol incorporating a CPR and the MoleMate scanning technique [42]. A CPR for head injured patients did not lead to a reduction in ED use of CT imaging [39], but a CPR for patients with blunt head and neck trauma and possible cervical spine fracture significantly decreased imaging without missing injuries [38].

## Discussion

The diagnostic CPRs evaluated in this review were found to have beneficial effects on process outcomes in some clinical conditions and in some cases had a positive effect on patient health. Though improvement in patient outcome, or increased efficiency of the diagnostic process without worsening patient outcomes, is the ultimate measure of effectiveness for diagnostic CPRs, few studies have primarily aimed to determine the effect of diagnostic CPRs, or diagnostic strategies incorporating CPRs, on patient outcomes. The majority of studies included in this review investigated more intermediate consequences of the use of the CPR, such as decisions to test or treat. Study methods, intervention and implementation details necessary for interpretation and safe application of the intervention were generally poorly reported. This non-transparency hinders attempts to replicate studies or their findings and erodes the value of the research in this area [49, 50].

The conclusions drawn in this review are based on a small number of studies, and, as a substantial number of these studies were categorised as high or unclear risk of bias on 3 or more domains of bias (one of which was performance bias), caution is advised in interpretation of their results. Further, the conclusions of many of the studies included in this review are likely to be limited by inadequate sample size [51]. Potential bias varied across studies for different clinical conditions, and it was often difficult to judge whether any bias would result in an over- or underestimation of intervention effect. The assessment of risk of bias relied upon the reporting of trials, and there was insufficient detail to confidently assess risk in many cases. Due to the nature of the interventions included in this review, which requires interaction with and interpretation by clinicians, blinding of clinicians was not possible. As such, clinicians’ prior expectations of effectiveness of the intervention were judged to have the potential to lead to bias either through disparity in other care that is administered to patients or by affecting clinicians’ decisions, which are an outcome in many studies. The risk arising from non-blinding of individuals assessing outcomes was judged unclear for most studies. This was due to either inadequate reporting or absence of blinding of independent data collectors or adjudicators.

The performance of the interventions evaluated in this review is likely to be dependent on the context in which the diagnostic strategy is implemented. For example, in a study of the effect of a CPR for predicting streptococcal infection conducted in general practice in the UK, discretionary application of the CPR decreased the severity of sore throat symptoms compared to a strategy of delayed antibiotic prescribing [18]. It is not clear how the prediction rule could affect resolution of sore throat, but it is possible that it helped to identify patients who would respond to antibiotics more accurately or quickly. In a setting where antibiotics are prescribed for sore throat more frequently and possibly earlier, the relative effect of the prediction rule on symptoms may be different. As another example, a diagnostic strategy incorporating a CPR and BNP testing may lead to less hospital admissions in a country where admissions for possible cardiac chest pain are common [36], but may have less effect in a situation where such admissions are infrequent.

Within and across clinical conditions, there was heterogeneity in the degree to which CPRs and diagnostic strategies incorporating CPRs were used and their output applied. The protocols of the included studies took one of two approaches to the use of the CPRs or diagnostic strategies: a pragmatic approach in which clinicians could decide whether or not to use the tool or an approach in which clinicians in the study were expected to use the CPR or were provided with the output from it. Further, there were varying degrees to which the clinician was required to follow the recommendation provided by the directive rules or strategies. In some studies, the subsequent treatment provided was dictated entirely by the CPR. In others, the clinician was ‘encouraged’ to follow the recommendations, and in others, clinicians could adopt or ignore the recommendations at their discretion. These variations not only may lead to differences in intervention effect but also have implications for transferring the research findings to clinical practice. Results from studies mandating use of a CPR and carefully monitoring its correct application may be different to results seen when the CPR is introduced in a situation where clinicians are given license to override its recommendations. It has been suggested that impact studies should assess both *actual* impact—impact when clinicians can use their discretion in following the CPR recommendations, and *potential* impact—measured by analysing the CPR-recommended decision regardless of implementation by the clinician [5]. This was done by two studies included in the review [29, 44]. In one of these studies [44], strictly following the CPR recommendations would have resulted in a treatment rate (antibiotic prescribing) of 31% as opposed to an actual treatment rate of 42% when clinicians could override the CPR recommendations (control group treatment rate 42.1 and 41.7% in the entire study cohort). In a secondary analysis of another included study, clinicians requested objective testing for 19 of 66 patients classified as low risk (and eligible for early discharge) by the care pathway including the CPR (none of the 19 patients had a MACE during follow-up). Had the recommendation of the care pathway been followed, the rate of early discharge would have increased from 40 to 53% [47]. Such information is likely to assist in the interpretation of impact study findings by informing of the interactions taking place between the clinician and the CPR and the reasons for any disagreements.

Though clear reporting of interventions is necessary for interpretation of study findings and safe replication of the intervention in practice [52], documentation of the interventions tested in the majority of the included studies was poor. This is similar to research on other complex interventions and non-pharmaceutical treatments [53, 54]. Furthermore, studies rarely stated how a diagnostic CPR is expected to alter outcomes relative to the alternate diagnostic strategy, making it difficult to judge the adequacy of the outcomes reported. For many of the included studies where the control intervention could broadly be described as usual care, no or minimal description of the test method or the criteria by which management decisions were made was provided. Usual care had various permutations ranging from what is termed ‘wild type’ or ‘care as it is now’, to a more regimented guideline-driven care [55, 56]. It is acknowledged that such strategies are internalised, likely complex, probably highly variable and nuanced and difficult to translate into a prescriptive format. However, lack of even mention of the tests performed makes it very difficult to interpret differences in trial outcomes and to judge generalisability. Furthermore, basic details about the process of implementation were infrequently reported, making it difficult to know whether failure to demonstrate an effect is more likely due to inadequate implementation of the experimental strategy, than lack of effect of the experimental intervention itself. Nor did the majority of studies provide information on which to judge the risk of behaviour change among clinicians in the control groups arising from knowledge of study conditions [56].

Despite the importance of patient-centred outcomes for informing decision-making, few studies included in this review report any patient outcomes, and fewer still report patient outcomes as the primary study outcome. Clinicians’ decisions to test or treat are the primary outcomes, and sometimes the only study outcome, in the majority of the included studies. There may be several explanations for this: randomised studies reporting patient outcomes are difficult, costly and time consuming to conduct; researchers may believe that patient management is a valid surrogate for health outcomes; or researchers may select outcomes that reflect the primary intention of many diagnostic CPRs to reduce testing or treatment. However, recent research suggests that it is not possible to infer the effects of a diagnostic test on patient outcomes based on how a test influences management decisions. In an analysis of a large sample of diagnostic randomised controlled trials, the effects of the index test on further diagnostic and therapeutic interventions did not correlate with the effects on patient outcomes [2]. This study also found that estimates of accuracy do not inform well about the clinical utility of diagnostic tests. Given the multitude of ways CPRs may affect patient outcomes [3, 4], improved accuracy or management decisions afforded by a CPR are neither a necessary requisite nor a guarantee for improving patient health. Though measurement of the effects of CPRs on patient management may be of some use for planning further evaluations of a CPR, and as part of a suite of outcome measures to assist in understanding the means by which a CPR may exert its effects, we argue that impact studies reporting only management decisions, or reporting management decisions without considering effects on patient outcomes, are insufficient to judge the clinical utility of diagnostic CPRs.

To our knowledge, this is the first review specifically of diagnostic CPRs across a range of clinical conditions. We are aware of one recently published systematic review evaluating the impact of diagnostic and prognostic CPRs (as a stand-alone tool) for conditions encountered in primary care [11]. Similarly, this review identified only a small number of studies evaluating the impact of CPRs in a limited number of clinical domains. Lack of evidence about the effect of use of diagnostic CPRs may be one factor contributing to their limited uptake into practice. Conventionally, CPRs are required to go through the stages of derivation, validation and impact analysis prior to full implementation in practice. This rigorous requirement is seemingly inconsistent with the situation for other diagnostic tests (such as point of care tests), which are frequently and fervently adopted into practice on the basis of demonstrated practical advantage or accuracy against a reference standard alone. Our review was limited to studies randomly allocating participants to care with or without a diagnostic CPR. However, it is likely, as examination of our table of excluded studies and the review of Wallace [11] suggests, that the use of non-randomised and uncontrolled study designs to assess the impact of diagnostic CPRs is reasonably common. This may reflect the practical and methodological challenges in performing randomised trials in the clinical setting particularly for conditions with potentially serious and often infrequent outcomes. While examining evaluations using non-randomised and observational designs would have contributed to the overall evidence base, only randomised controlled trials can rule out the possibility that an observed association between an intervention and outcome is caused by a third factor linked to both. The findings of our review, that CPRs reduce prescribing and test ordering for some conditions, are also generally consistent with existing research evaluating clinical decision support tools more broadly, which has found that some systems can improve test ordering and antibiotic prescribing behaviour [6–8].

Our review has limitations. Because of the large number of titles and abstracts retrieved in the searches, only one reviewer performed screening of titles and abstracts, with a second reviewer screening only a proportion. Therefore, some studies may have been overlooked. However, screening of systematic reviews of clinical decision support systems, reference checks and forward searches minimised the possibility that eligible studies were missed. The presence of study publication bias in this review is possible. For instance, many of the CPRs were tested by the researchers who developed the CPR and thus may be more likely to submit studies with positive results for publication. In reporting whether a study described components of the interventions, we determined only whether a description was present, rather than providing a judgment about the adequacy of the description. Consequently, the review is likely to overestimate the reporting quality of the included studies, as components were judged to have been described even if only partially so or with little detail. Furthermore, the criteria assessed were considered by the authors to be the minimum essential to the reporting of intervention content. To properly appraise reporting quality of impact studies, more criteria should be considered.

## Conclusions

This review provides insight into the current status of research evaluating the impact of diagnostic CPRs and provides information that may assist clinicians and policymakers’ decisions regarding the application of these tools. This review found that diagnostic CPRs improve process of care measures for some clinical conditions and in some cases improved or maintained patient health while providing other benefits. However, this conclusion is based on a small number of studies, many of which are judged to be at high or unclear risk of bias and is likely to be context dependent. It is apparent from this review that future impact studies need to be more carefully designed and conducted and more thoroughly reported. Consideration of the many mechanisms by which a CPR may alter outcomes during the trial design stage should guide the nature and number of outcomes measured and facilitate understanding of why particular effects are observed. Use of a framework such as that developed by Ferrante di Ruffano and colleagues [3] may assist firstly in identifying the means by which a CPR may alter the existing diagnostic pathway and secondly to consideration of the full range of direct and downstream outcomes that should be measured. Furthermore, reporting of such studies should be improved to assist interpretation and replication in practice. Establishing benefit to patient health or showing that patient health can be maintained while providing other benefits should be the priority of impact evaluations of diagnostic prediction rules.

## Additional files

Additional file 1: PRISMA Statement. (DOC 63 kb)

Additional file 2: Electronic database search strategies. (DOC 32 kb)

Additional file 3: Full-text studies excluded from the review with reason. (DOC 267 kb)

Additional file 4: Primary and secondary outcomes reported in the included studies. (DOC 61 kb)

Additional file 5: Risk of bias graph. Review authors’ judgments about each risk of bias domain for each included study. (DOC 190 kb)

Additional file 6: The performance of included studies against the minimum required elements for reporting of diagnostic strategies and implementation methods. (DOC 242 kb)

Additional file 7: Results of studies of diagnostic CPRs for suspected Group A *Streptococcus* throat infection by outcome. Results of studies of diagnostic CPRs for suspected acute appendicitis by outcome. Results of studies of diagnostic CPRs for suspected serious bacterial infection in children with fever. Results of studies of diagnostic CPRs for suspected acute coronary syndrome by outcome. Results of studies of diagnostic CPRs for suspected (bacterial) pneumonia by outcome. Results of studies of diagnostic CPRs for suspected ankle or mid-foot fracture by outcome. Results of single studies of diagnostic CPRs for different clinical conditions. (DOC 538 kb)

## Abbreviation

CPR: Clinical prediction rule
