# Supplementary material for: Systematic review of the effects of care provided with and without diagnostic clinical prediction rules
Source: Diagn Progn Res. 2017 Apr 26;1:13. doi: 10.1186/s41512-017-0013-2 (PMC6460683; doi:10.1186/s41512-017-0013-2)
Supplement: Supplementary file 2 — Electronic database search strategies. (DOC 32 kb) [file 41512_2017_13_MOESM2_ESM.doc]

**Additional File 2**

Electronic database search strategies

| Ovid MEDLINE(R) In-Process & Other Non-Indexed Citations and Ovid MEDLINE(R) 1946 to 15/09/16. | 1. Randomized controlled trial.pt.  2. Controlled clinical trial.pt.  3. Randomized.ab.  4. Placebo.ab.  5. Clinical trials as topic.sh.  6. Randomly.ab.  7. Trial.ti.  8. 1 or 2 or 3 or 4 or 5 or 6 or 7  9. exp animals/ not humans.sh.  10. 8 not 9  11. *Decision Support Systems, Clinical/  12. Rule*.ti.  13. (rule* adj3 (decision OR clinical OR diagnos* OR predict*).ti,ab.  14. Score*.ti.  15. (score* adj3 (decision OR clinical OR diagnos* OR predict* OR risk).ti,ab.  16. ((aid or model) adj3 (clinical or decision or diagnos* or predict*).ti,ab.  17. ((guide or algorithm or protocol) adj2 (diagnos* or decision or clinical or predict*)).ti,ab.  18. 11 or 12 or 13 or 14 or 15 or16 or 17  19. 18 and 10 |
| --- | --- |
| Cochrane Central Register of Controlled Trials (CENTRAL, Issue 8, 2016) in the Cochrane Library. Searched 16/09/2016. | (score* OR rule* OR ((protocol OR aid OR algorithm OR tool OR instrument) near/2 (diagnos* OR decision OR clinical OR predict*)) ):ti |
