# Supplementary material for: Systematic review of the effects of care provided with and without diagnostic clinical prediction rules
Source: Diagn Progn Res. 2017 Apr 26;1:13. doi: 10.1186/s41512-017-0013-2 (PMC6460683; doi:10.1186/s41512-017-0013-2)
Supplement: Supplementary file 3 — Full-text studies excluded from the review with reason. (DOC 267 kb) [file 41512_2017_13_MOESM3_ESM.doc]

**Additional File 3**

Full text studies excluded from the review with reason

| **Reference** | **Comment** |
| --- | --- |
| Not a study of randomised allocation to care with and without a prediction rule | |
| | Ackerman SL, Gonzales R, Stahl MS, Metlay JP. One size does not fit all: evaluating an intervention to reduce antibiotic prescribing for acute bronchitis. BMC Health Serv Res. 2013;13:462. PubMed PMID: 24188573. Pubmed Central PMCID: 4228248. |  | | --- | --- | | Not a randomised comparison |
| | Adams ID, Chan M, Clifford PC, Cooke WM, Dallos V, Dombal FT, et al. Computer aided diagnosis of acute abdominal pain: a multicentre study. British Medical Journal [Internet]. 1986; 293(6550):[800-4 pp.]. |  | | --- | --- | | Before after study |
| | Ammirati F, Colivicchi F, Santini M. Diagnosing syncope in clinical practice. Implementation of a simplified diagnostic algorithm in a multicentre prospective trial - the OESIL 2 study (Osservatorio Epidemiologico della Sincope nel Lazio). European Heart Journal. 2000;21(11):935-40. PubMed PMID: 10806018. |  | | --- | --- | | Not a controlled study |
| Anonymous. Early data suggest new protocol to risk-stratify chest pain patients, potentially preserving resources without compromising safety. ED Management. 2015;27(5):49-52. PubMed PMID: 25932495. | Not a controlled study |
| | Beltrán MA, Villar MR, Cruces KS. [Application of a diagnostic score for appendicitis by health-related non-physician professionals]. Revista médica de Chile [Internet]. 2006; 134(1):[39-47 pp.] |  | | --- | --- | |  | | Not a randomised comparison |
| Bajaj RR, Goodman SG, Yan RT, Bagnall AJ, Gyenes G, Welsh RC, et al. Treatment and outcomes of patients with suspected acute coronary syndromes in relation to initial diagnostic impressions (insights from the Canadian Global Registry of Acute Coronary Events [GRACE] and Canadian Registry of Acute Coronary Events [CANRACE]). American Journal of Cardiology. 2013;111(2):202-7. PubMed PMID: 23122889. | Not a randomised comparison |
| | Bessen T, Clark R, Shakib S, Hughes G. A multifaceted strategy for implementation of the Ottawa ankle rules in two emergency departments. BMJ. 2009;339:b3056. PubMed PMID: 19675080. Pubmed Central PMCID: 2726279. |  | | --- | --- | | Before after study |
| | Bressan S, editor Implementation of PECARN decision rule for children with minor head injury in the pediatric emergency department. Mediterranean Emergency Medicine Congress (VI); 2011. |  | | --- | --- | | Before after study |
| Boutis K, Grootendorst P, Willan A, Plint AC, Babyn P, Brison RJ, et al. Effect of the Low Risk Ankle Rule on the frequency of radiography in children with ankle injuries. CMAJ : Canadian Medical Association journal = journal de l'Association medicale canadienne. 2013 Oct 15;185(15):E731-8. PubMed PMID: 23939215. Pubmed Central PMCID: 3796622. | Not a randomised comparison. An interrupted time series study with matched control |
| | Brand DA, Frazier WH, Kohlhepp WC, Shea KM, Hoefer AM, Ecker MD, et al. A protocol for selecting patients with injured extremities who need x-rays. N Engl J Med. 1982 Feb 11;306(6):333-9. PubMed PMID: 7054709. |  | | --- | --- | | Not a controlled study |
| | Broekhuizen BD, Sachs A, Janssen K, Geersing GJ, Moons K, Hoes A, et al. Does a decision aid help physicians to detect chronic obstructive pulmonary disease? The British journal of general practice : the journal of the Royal College of General Practitioners. 2011 Oct;61(591):e674-9. PubMed PMID: 22152850. Pubmed Central PMCID: 3177137. |  | | --- | --- | | Not a randomised comparison. Study of the incremental value of the prediction rule |
| | Cameron C, Naylor CD. No impact from active dissemination of the Ottawa Ankle Rules: further evidence of the need for local implementation of practice guidelines. Cmaj [Internet]. 1999; 160(8):[1165-8 pp.]. |  | | --- | --- | | Not a randomised comparison. Before after study of the effect of an active dissemination strategy. Investigators compared use of ankle radiography in hospitals receiving an educational intervention with some or no use of the OARs with hospitals who declined the dissemination strategy (already using the OARs). |
| | Casey JR, Block S, Puthoor P, Hedrick J, Almudevar A, Pichichero ME. A simple scoring system to improve clinical assessment of acute otitis media. Clinical pediatrics [Internet]. 2011; 50(7):[623-9 pp.]. |  | | --- | --- | | Not a controlled study |
| | Christian F, Christian GP. A simple scoring system to reduce the negative appendicectomy rate. Annals of the Royal College of Surgeons of England [Internet]. 1992; 74(4):[281-5 pp.]. |  | | --- | --- | | Not a randomised comparison |
| | Courtney DM, Kline JA. Prospective use of a clinical decision rule to identify pulmonary embolism as likely cause of outpatient cardiac arrest. Resuscitation [Internet]. 2005; 65(1):[57-64 pp.]. |  | | --- | --- | | Not a controlled study |
| | den Exter PL, Gomez V, Jimenez D, Trujillo-Santos J, Muriel A, Huisman MV, et al. A clinical prognostic model for the identification of low-risk patients with acute symptomatic pulmonary embolism and active cancer. Chest. 2013;143(1):138-45. PubMed PMID: 22814859. |  | | --- | --- | | Not a controlled study |
| | Dobbs F. A scoring system for predicting group A streptococcal throat infection. British Journal of General Practice. 1996;46(409):461-4. PubMed PMID: 8949324. Pubmed Central PMCID: PMC1239715. |  | | --- | --- | | Not a controlled study |
| | Dombal FT, Leaper DJ, Staniland JR, McCann AP, Horrocks JC. Computer-aided diagnosis of acute abdominal pain. British medical journal [Internet]. 1972; 2(5804):[9-13 pp.]. |  | | --- | --- | | Not a controlled study |
| | Drescher FS, Chandrika S, Weir ID, Weintraub JT, Berman L, Lee R, et al. Effectiveness and acceptability of a computerized decision support system using modified Wells criteria for evaluation of suspected pulmonary embolism. Ann Emerg Med. 2011 Jun;57(6):613-21. PubMed PMID: 21050624. |  | | --- | --- | | Not a randomised comparison |
| | Enochsson L, Gudbjartsson T, Hellberg A, Rudberg C, Wenner J, Ringqvist I, et al. The Fenyö-Lindberg scoring system for appendicitis increases positive predictive value in fertile women--a prospective study in 455 patients randomized to either laparoscopic or open appendectomy. Surgical endoscopy [Internet]. 2004; 18(10):[1509-13 pp.]. |  | | --- | --- | | Not a controlled study |
| | Eccles M, Steen N, Grimshaw J, Thomas L, McNamee P, Soutter J, et al. Effect of audit and feedback, and reminder messages on primary-care radiology referrals: a randomised trial. Lancet. 2001 May 5;357(9266):1406-9. PubMed PMID: 11356439. |  | | --- | --- | | No control group without Guideline.Guideline does not appear to include a CPR. |
| | Gonzales R, Aagaard EM, Camargo CA, Jr., Ma OJ, Plautz M, Maselli JH, et al. C-reactive protein testing does not decrease antibiotic use for acute cough illness when compared to a clinical algorithm. Journal of Emergency Medicine. 2011;41(1):1-7. PubMed PMID: 19095403. |  | | --- | --- | | No control group without CPR |
| | Green L, Mehr DR. What alters physicians' decisions to admit to the coronary care unit? J Fam Pract. 1997 Sep;45(3):219-26. PubMed PMID: 9300001. |  | | --- | --- | | Interrupted time series |
| | Goldman L, Cook EF, Brand DA, Lee TH, Rouan GW, Weisberg MC, et al. A computer protocol to predict myocardial infarction in emergency department patients with chest pain. N Engl J Med. 1988 Mar 31;318(13):797-803. PubMed PMID: 3280998. |  | | --- | --- | | Not a controlled study |
| | Holroyd BR, Wilson D, Rowe BH, Mayes DC, Noseworthy T. Uptake of validated clinical practice guidelines: experience with implementing the Ottawa Ankle Rules. The American journal of emergency medicine [Internet]. 2004; 22(3):[149-55 pp.]. |  | | --- | --- | | Not a randomised comparison |
| | Harizman N, Oliveira C, Chiang A, Tello C, Marmor M, Ritch R, et al. The ISNT rule and differentiation of normal from glaucomatous eyes. Archives of ophthalmology [Internet]. 2006; 124(11):[1579-83 pp.]. |  | | --- | --- | | Not a controlled study |
| | Hsu P, Lam LT, Browne G. The pulmonary index score as a clinical assessment tool for acute childhood asthma. Annals of allergy, asthma & immunology : official publication of the American College of Allergy, Asthma, & Immunology [Internet]. 2010; 105(6):[425-9 pp.]. |  | | --- | --- | | Not a randomised comparison |
| | Iapichino G, Mistraletti G, Corbella D, Bassi G, Borotto E, Miranda DR, et al. Scoring system for the selection of high-risk patients in the intensive care unit. Critical care medicine [Internet]. 2006; 34(4):[1039-43 pp.]. |  | | --- | --- | | Not a randomised comparison |
| | Jacobs AA. Clinical prediction of pneumonia. Annals of Internal Medicine. 1991;1114(4):428. |  | | --- | --- | | Letter to editor |
| | Kerr D, Bradshaw L, Kelly AM. Implementation of the Canadian C-spine rule reduces cervical spine x-ray rate for alert patients with potential neck injury. J Emerg Med. 2005 Feb;28(2):127-31. PubMed PMID: 15707805. |  | | --- | --- | | Not a randomised comparison |
| | Kilroy DA, Ireland S, Reid P, Goodacre S, Morris F. Emergency department investigation of deep vein thrombosis. Emergency medicine journal : EMJ. 2003 Jan;20(1):29-32. PubMed PMID: 12533363. Pubmed Central PMCID: 1726005. |  | | --- | --- | | Not a controlled study |
| | Kec RM, Richman PB, Szucs PA, Mandell M, Eskin B. Can emergency department triage nurses appropriately utilize the Ottawa Knee Rules to order radiographs?-An implementation trial. Academic Emergency Medicine. 2003;10(2):146-50. PubMed PMID: 12574012. |  | | --- | --- | | Not a controlled study |
| | Khan I, Rehman AU. Application of alvarado scoring system in diagnosis of acute appendicitis. Journal of Ayub Medical College Abbottabad [Internet]. 2005; 17(3):[41-4 pp.]. |  | | --- | --- | | Not a controlled study |
| | Kotowycz MA, Cosman TL, Tartaglia C, Afzal R, Syal RP, Natarajan MK. Safety and feasibility of early hospital discharge in ST-segment elevation myocardial infarction--a prospective and randomized trial in low-risk primary percutaneous coronary intervention patients (the Safe-Depart Trial). American Heart Journal. 2010;159(1):117.e1-6. PubMed PMID: 20102876. |  | | --- | --- | | Not a randomised comparison (diagnostic CPR used for assessing inclusion in the study) |
| | Lopez PP, Cohn SM, Popkin CA, Jackowski J, Michalek JE, Appendicitis Diagnostic G. The use of a computed tomography scan to rule out appendicitis in women of childbearing age is as accurate as clinical examination: a prospective randomized trial. American Surgeon. 2007;73(12):1232-6. PubMed PMID: 18186378. |  | | --- | --- | | Not a randomised comparison (diagnostic CPR used for assessing inclusion in the study) |
| | Leddy JJ, Kesari A, Smolinski RJ. Implementation of the Ottawa ankle rule in a university sports medicine centre. Medicine and science in sports and exercise. 2002 Jan;34(1):57-62. PubMed PMID: 11782648. |  | | --- | --- | | Not a controlled study |
| | Lee TH, Pearson SD, Johnson PA, Garcia TB, Weisberg MC, Guadagnoli E, et al. Failure of information as an intervention to modify clinical management. A time-series trial in patients with acute chest pain. Ann Intern Med. 1995 Mar 15;122(6):434-7. PubMed PMID: 7856992. |  | | --- | --- | | Not a randomised comparison |
| | Matloob SA, Roach J, Marcus HJ, O'Neill K, Nair R. Evaluation of the impact of the Canadian subarachnoid haemorrhage clinical decision rules on British practice. British journal of neurosurgery. 2013 Oct;27(5):603-6. PubMed PMID: 23730979. |  | | --- | --- | | Not a controlled study |
| | McAdam WA, Brock BM, Armitage T, Davenport P, Chan M, de Dombal FT. Twelve years' experience of computer-aided diagnosis in a district general hospital. Ann R Coll Surg Engl. 1990 Mar;72(2):140-6. PubMed PMID: 2185682. Pubmed Central PMCID: 2499113. |  | | --- | --- | | Interrupted time series study |
| McIsaac WJ, Kellner JD, Aufricht P, Vanjaka A, Low DE. Empirical validation of guidelines for the management of pharyngitis in children and adults. JAMA. 2004 Apr 7;291(13):1587-95. PubMed PMID: 15069046. | Not a randomised comparison |
| | Mortola GP, Arnulfo G, Reboa G, Pitto G, Masini R, DiSomma C, et al. Clinical application of a computerized diagnostic aid in the initial evaluation of 250 outpatients with gastrointestinal complaints. Journal of Clinical Computing. 1987;16(3-4):93-103. PubMed PMID: 10302545. |  | | --- | --- | | Not a randomised comparison |
| Mahler SA, Riley RF, Russell GB, Hiestand BC, Hoekstra JW, Lefebvre CW, et al. Adherence to an Accelerated Diagnostic Protocol for Chest Pain: Secondary Analysis of the HEART Pathway Randomized Trial. Academic Emergency Medicine. 2016;23(1):70-7. PubMed PMID: 26720295. Pubmed Central PMCID: NIHMS734559 | Not a controlled study |
| | Mainous AG, 3rd, Lambourne CA, Nietert PJ. Impact of a clinical decision support system on antibiotic prescribing for acute respiratory infections in primary care: quasi-experimental trial. Journal of the American Medical Informatics Association. 2013;20(2):317-24. PubMed PMID: 22759620. Pubmed Central PMCID: PMC3638170 |  | | --- | --- | | Not a randomised comparison |
| | Man E, Simonka Z, Varga A, Rarosi F, Lazar G. Impact of the Alvarado score on the diagnosis of acute appendicitis: comparing clinical judgment, Alvarado score, and a new modified score in suspected appendicitis: a prospective, randomized clinical trial. Surg Endosc. 2014 Aug;28(8):2398-405. PubMed PMID: 24705731. |  | | --- | --- | | Not a randomised comparison |
| | Naschitz JE, Rosner I, Rozenbaum M, Naschitz S, Musafia-Priselac R, Shaviv N, et al. The head-up tilt test with haemodynamic instability score in diagnosing chronic fatigue syndrome. QJM : monthly journal of the Association of Physicians [Internet]. 2003; 96(2):[133-42 pp.]. Available from: http://onlinelibrary.wiley.com/o/cochrane/clcentral/articles/734/CN-00422734/frame.html. |  | | --- | --- | | Not a controlled study |
| | Ohmann C, Franke C, Yang Q. Clinical benefit of a diagnostic score for appendicitis: results of a prospective interventional study. German Study Group of Acute Abdominal Pain. Arch Surg. 1999 Sep;134(9):993-6. PubMed PMID: 10487595. |  | | --- | --- | | Before after study |
| | Owen TD, Williams H, Stiff G, Jenkinson LR, Rees BI. Evaluation of the Alvarado score in acute appendicitis. J R Soc Med. 1992 Feb;85(2):87-8. PubMed PMID: 1489366. Pubmed Central PMCID: 1294889. |  | | --- | --- | | Not a randomised comparison |
| | Pitt E, Pedley DK, Nelson A, Cumming M, Johnston M. Removal of C-spine protection by A&E triage nurses: a prospective trial of a clinical decision making instrument. Emergency Medicine Journal. 2006;23(3):214-5. PubMed PMID: 16498160. Pubmed Central PMCID: PMC2464447. |  | | --- | --- | | Not a controlled study |
| | Poses RM, Cebul RD, Wigton RS, Centor RM, Collins M, Fleischli G. Controlled trial using computerized feedback to improve physicians' diagnostic judgments. Academic Medicine. 1992;67(5):345-7. PubMed PMID: 1575873. |  | | --- | --- | | Non randomised before after study with concurrent controls |
| | Pozen MW, D'Agostino RB, Mitchell JB, Rosenfeld DM, Guglielmino JT, Schwartz ML, et al. The usefulness of a predictive instrument to reduce inappropriate admissions to the coronary care unit. Annals of internal medicine [Internet]. 1980; 92(2 Pt 1):[238-42 pp.]. |  | | --- | --- | | Interrupted time series |
| | Pozen MW, D'Agostino RB, Selker HP, Sytkowski PA, Hood WB, Jr. A predictive instrument to improve coronary-care-unit admission practices in acute ischemic heart disease. A prospective multicenter clinical trial. New England Journal of Medicine. 1984;310(20):1273-8. PubMed PMID: 6371525. |  | | --- | --- | | Not a randomised comparison (allocated by alternate months or 6 month period)/reference standard for diagnosis not current |
| | Roy PM, Durieux P, Gillaizeau F, Legall C, Armand-Perroux A, Martino L, et al. A computerized handheld decision-support system to improve pulmonary embolism diagnosis: a randomized trial. Annals of Internal Medicine. 2009;151(10):677-86. PubMed PMID: 19920268. |  | | --- | --- | | Not a randomised comparison of CPR vs no CPR. CPR used in both study groups as either hand held computerised form or paper form. |
| | Roberts RR, Zalenski RJ, Mensah EK, Rydman RJ, Ciavarella G, Gussow L, et al. Costs of an emergency department-based accelerated diagnostic protocol vs hospitalization in patients with chest pain: a randomized controlled trial. JAMA. 1997;278(20):1670-6. PubMed PMID: 9388086. |  | | --- | --- | | Not a controlled study |
| | | Reilly BM, Evans AT, Schaider JJ, Das K, Calvin JE, Moran LA, et al. Impact of a clinical decision rule on hospital triage of patients with suspected acute cardiac ischemia in the emergency department. JAMA. 2002 Jul 17;288(3):342-50. PubMed PMID: 12117399. |  | | --- | --- | |  | | --- | --- | --- | --- | | Interrupted time series |
| | Righini M, Le Gal G, Aujesky D, Roy PM, Sanchez O, Verschuren F, et al. Diagnosis of pulmonary embolism by multidetector CT alone or combined with venous ultrasonography of the leg: a randomised non-inferiority trial. Lancet. 2008 Apr 19;371(9621):1343-52. PubMed PMID: 18424324. |  | | --- | --- | | Not a randomised comparison of CPR vs no CPR. CPR used in all participants who were then randomised to different sequences of testing |
| | Reilly BM, Evans AT, Schaider JJ, Wang Y. Triage of patients with chest pain in the emergency department: a comparative study of physicians' decisions. Am J Med. 2002 Feb 1;112(2):95-103. PubMed PMID: 11835946. |  | | --- | --- | | Not a randomised comparison |
| | Sarasin FP, Reymond JM, Griffith JL, Beshansky JR, Schifferli JA, Unger PF, et al. Impact of the acute cardiac ischemia time-insensitive predictive instrument (ACI-TIPI) on the speed of triage decision making for emergency department patients presenting with chest pain: a controlled clinical trial. Journal of general internal medicine [Internet]. 1994; 9(4):[187-94 pp.]. |  | | --- | --- | | No control group without CPR |
| | Scheye T, Vanneuville G. [Trial of a diagnostic score in painful abdominal syndromes suggestive of appendicitis in children over 3]. Journal de Chirurgie. 1988;125(3):166-9. PubMed PMID: 3372603. |  | | --- | --- | | Not a randomised comparison (diagnostic CPR used for assessing inclusion in the study) |
| | Selker HP, Beshansky JR, Griffith JL. Use of the electrocardiograph-based thrombolytic predictive instrument to assist thrombolytic and reperfusion therapy for acute myocardial infarction. A multicenter, randomized, controlled, clinical effectiveness trial. Annals of internal medicine [Internet]. 2002; 137(2):[87-95 pp.]. |  | | --- | --- | | Interrupted time series |
| | Selker HP, Beshansky JR, Griffith JL, Aufderheide TP, Ballin DS, Bernard SA, et al. Use of the acute cardiac ischemia time-insensitive predictive instrument (ACI-TIPI) to assist with triage of patients with chest pain or other symptoms suggestive of acute cardiac ischemia. A multicenter, controlled clinical trial. Annals of Internal Medicine. 1998;129(11):845-55. PubMed PMID: 9867725. |  | | --- | --- | | Not a controlled study |
| | Selker HP, Beshansky JR, Ruthazer R, Sheehan PR, Sayah AJ, Atkins JM, et al. Emergency medical service predictive instrument-aided diagnosis and treatment of acute coronary syndromes and ST-segment elevation myocardial infarction in the IMMEDIATE trial. Prehospital Emergency Care. 2011;15(2):139-48. PubMed PMID: 21366431. |  | | --- | --- | | Not a randomised comparison |
| | Stiell I, Wells G, Laupacis A, Brison R, Verbeek R, Vandemheen K, et al. Multicentre trial to introduce the Ottawa ankle rules for use of radiography in acute ankle injuries. Multicentre Ankle Rule Study Group. BMJ. 1995;311(7005):594-7. PubMed PMID: 7663253. Pubmed Central PMCID: PMC2550661. |  | | --- | --- | | Interrupted time series |
| | Sutton GC. How accurate is computer-aided diagnosis? Lancet [Internet]. 1989; 2(8668):[905-8 pp.]. |  | | --- | --- | | Not a randomised comparison |
| | Stiell IG, McKnight RD, Greenberg GH, McDowell I, Nair RC, Wells GA, et al. Implementation of the Ottawa ankle rules. JAMA. 1994 Mar 16;271(11):827-32. PubMed PMID: 8114236. |  | | --- | --- | | Non randomised before after study with concurrent controls |
| | Stiell IG, Wells GA, Hoag RH, Sivilotti ML, Cacciotti TF, Verbeek PR, et al. Implementation of the Ottawa Knee Rule for the use of radiography in acute knee injuries. JAMA. 1997;278(23):2075-9. PubMed PMID: 9403421. |  | | --- | --- | | Non randomised before after study with concurrent controls |
| | Singh S, Nosyk B, Sun H, Christenson JM, Innes G, Anis AH. Value of information of a clinical prediction rule: informing the efficient use of healthcare and health research resources. Int J Technol Assess Health Care. 2008 Winter;24(1):112-9. PubMed PMID: 18218176. |  | | --- | --- | | Decision analytic modelling study |
| | Wells PS, Hirsh J, Anderson DR, Lensing AW, Foster G, Kearon C, et al. A simple clinical model for the diagnosis of deep-vein thrombosis combined with impedance plethysmography: potential for an improvement in the diagnostic process. J Intern Med. 1998 Jan;243(1):15-23. PubMed PMID: 9487327. |  | | --- | --- | | Not a controlled study |
| | Woolley SL, Bernstein JM, Davidson JA, Smith DR. Sore throat in adults--does the introduction of a clinical scoring system improve the management of these patients in a secondary care setting? The Journal of laryngology and otology. 2005 Jul;119(7):550-5. PubMed PMID: 16175981. |  | | --- | --- | | Before after study |
| Weingarten S, Ermann B, Bolus R, Riedinger MS, Rubin H, Green A, et al. Early "step-down" transfer of low-risk patients with chest pain. A controlled interventional trial. Annals of Internal Medicine. 1990;113(4):283-9. PubMed PMID: 2115754. |  |
| | Wells PS, Anderson DR, Rodger M, Forgie M, Kearon C, Dreyer J, et al. Evaluation of D-dimer in the diagnosis of suspected deep-vein thrombosis. New England Journal of Medicine. 2003;349(13):1227-35. PubMed PMID: 14507948. |  | | --- | --- | | Not a randomised comparison of CPR versus no CPR (CPR applied to all then randomised to different management strategy based on output of CPR) |
| | Wilson PD, Horrocks JC, Lyndon PJ, Yeung CK, Page RE, Dombal FT. Simplified computer-aided diagnosis of acute abdominal pain. British medical journal [Internet]. 1975; 2(5962):[73-5 pp.]. |  | | --- | --- | | Not a controlled study |
| | Winn RD, Laura S, Douglas C, Davidson P, Gani JS. Protocol-based approach to suspected appendicitis, incorporating the Alvarado score and outpatient antibiotics. ANZ J Surg. 2004 May;74(5):324-9. PubMed PMID: 15144250. |  | | --- | --- | | Not a randomised comparison |
| | Wilson EC, Emery JD, Kinmonth AL, Prevost AT, Morris HC, Humphrys E, et al. The cost-effectiveness of a novel SIAscopic diagnostic aid for the management of pigmented skin lesions in primary care: a decision-analytic model. Value in Health. 2013;16(2):356-66. PubMed PMID: 23538188. |  | | --- | --- | | Decision analytic modelling economic study incorporating information from RCT included in the review |
| | Westfall JM, Van Vorst RF, McGloin J, Selker HP. Triage and diagnosis of chest pain in rural hospitals: implementation of the ACI-TIPI in the High Plains Research Network. Ann Fam Med. 2006 Mar-Apr;4(2):153-8. PubMed PMID: 16569719. Pubmed Central PMCID: 1467005. |  | | --- | --- | | Not a randomised comparison |
| Intervention does not include a diagnostic prediction rule or the prediction rule is intended to facilitate clinician and patient shared decision making | |
| | Achaval S, Fraenkel L, Volk RJ, Cox V, Suarez-Almazor ME. Impact of educational and patient decision aids on decisional conflict associated with total knee arthroplasty. Arthritis care & research [Internet]. 2012; 64(2):[229-37 pp.]. |  | | --- | --- | | Intervention is a patient decision aid not a diagnostic CPR for clinician use only |
| | Anderson RT, Montori VM, Shah ND, Ting HH, Pencille LJ, Demers M, et al. Effectiveness of the Chest Pain Choice decision aid in emergency department patients with low-risk chest pain: study protocol for a multicenter randomized trial. Trials. 2014;15:166. PubMed PMID: 24884807. Pubmed Central PMCID: 4031497. |  | | --- | --- | | Protocol for a study where the intervention is a patient decision aid not a diagnostic CPR for clinician use only |
| | Bell LM, Grundmeier R, Localio R, Zorc J, Fiks AG, Zhang X, et al. Electronic health record-based decision support to improve asthma care: a cluster-randomized trial. Pediatrics. 2010;125(4):e770-7. PubMed PMID: 20231191. |  | | --- | --- | | Intervention does not include a diagnostic CPR |
| | Bates DW, Kuperman GJ, Rittenberg E, Teich JM, Fiskio J, Ma'luf N, et al. A randomized trial of a computer-based intervention to reduce utilization of redundant laboratory tests. Am J Med. 1999 Feb;106(2):144-50. PubMed PMID: 10230742. |  | | --- | --- | | Intervention does not include a diagnostic CPR |
| Bollestad M, Grude N, Lindbaek M. A randomized controlled trial of a diagnostic algorithm for symptoms of uncomplicated cystitis at an out-of-hours service. Scandinavian Journal of Primary Health Care. 2015;33(2):57-64. PubMed PMID: 25961367. | Intervention does not include a diagnostic CPR |
| | Bourgeois FC, Linder J, Johnson SA, Co JP, Fiskio J, Ferris TG. Impact of a computerized template on antibiotic prescribing for acute respiratory infections in children and adolescents. Clin Pediatr (Phila). 2010 Oct;49(10):976-83. PubMed PMID: 20724348. |  | | --- | --- | | Intervention does not include a diagnostic CPR |
| | Christakis DA, Zimmerman FJ, Wright JA, Garrison MM, Rivara FP, Davis RL. A randomized controlled trial of point-of-care evidence to improve the antibiotic prescribing practices for otitis media in children. Pediatrics. 2001;107(2):E15. PubMed PMID: 11158489. |  | | --- | --- | | Intervention does not include a diagnostic CPR |
| | Carroll AE, Biondich P, Anand V, Dugan TM, Downs SM. A randomized controlled trial of screening for maternal depression with a clinical decision support system. Journal of the American Medical Informatics Association. 2013;20(2):311-6. PubMed PMID: 22744960. |  | | --- | --- | | Intervention does not include a diagnostic CPR |
| | Chang AB, Robertson CF, van Asperen PP, Glasgow NJ, Masters IB, Teoh L, et al. A cough algorithm for chronic cough in children: a multicenter, randomized controlled study. Pediatrics. 2013;131(5):e1576-83. PubMed PMID: 23610200. |  | | --- | --- | | Intervention does not include a diagnostic CPR |
| | Cheyne H, Hundley V, Dowding D, Bland JM, McNamee P, Greer I, et al. Effects of algorithm for diagnosis of active labour: cluster randomised trial. BMJ (Clinical research ed) [Internet]. 2008; 337:[a2396 p.]. |  | | --- | --- | | Intervention does not include a diagnostic CPR as defined by review (predictors identified by expert opinion and literature review not multivariable statistical analysis) |
| | Clayton TC, Lubsen J, Pocock SJ, Vokó Z, Kirwan BA, Fox KA, et al. Risk score for predicting death, myocardial infarction, and stroke in patients with stable angina, based on a large randomised trial cohort of patients. BMJ (Clinical research ed) [Internet]. 2005; 331(7521):[869 p.]. |  | | --- | --- | | Intervention does not include a diagnostic CPR |
| | Carroll AE, Bauer NS, Dugan TM, Anand V, Saha C, Downs SM. Use of a computerized decision aid for ADHD diagnosis: a randomized controlled trial. Pediatrics. 2013 Sep;132(3):e623-9. PubMed PMID: 23958768. Pubmed Central PMCID: 3876764. |  | | --- | --- | | Intervention does not include a diagnostic CPR |
| | Chang AB, Robertson CF, van Asperen PP, Glasgow NJ, Masters IB, Teoh L, et al. A cough algorithm for chronic cough in children: a multicenter, randomized controlled study. Pediatrics. 2013;131(5):e1576-83. PubMed PMID: 23610200. |  | | --- | --- | | Intervention does not include a diagnostic CPR |
| | Coutinho Storti F, Moffa PJ, Uchida AH, Hueb WA, Machado Cesar LA, Ferreira BM, et al. New prognostic score for stable coronary disease evaluation. Arquivos Brasileiros de Cardiologia. 2011;96(5):411-8. PubMed PMID: 21503388. |  | | --- | --- | | Intervention does not include a diagnostic CPR |
| | Del Mar CB, Green AC. Aid to diagnosis of melanoma in primary medical care. BMJ. 1995;310(6978):492-5. PubMed PMID: 7888887. Pubmed Central PMCID: PMC2548872. |  | | --- | --- | | Intervention does not include a diagnostic CPR as defined by review (predictors identified by expert opinion not multivariable statistical analysis) |
| Dexheimer JW, Abramo TJ, Arnold DH, Johnson K, Shyr Y, Ye F, et al. Implementation and evaluation of an integrated computerized asthma management system in a pediatric emergency department: a randomized clinical trial. Int J Med Inform. 2014 Nov;83(11):805-13. PubMed PMID: 25174321. | Intervention does not incorporate a diagnostic CPR |
| | Emmett CL, Montgomery AA, Peters TJ, Fahey T. Three-year follow-up of a factorial randomised controlled trial of two decision aids for newly diagnosed hypertensive patients. British Journal of General Practice [Internet]. 2005; 55(516):[551-3 pp.]. |  | | --- | --- | | Intervention does not include a diagnostic CPR |
| | English DR, Burton RC, del Mar CB, Donovan RJ, Ireland PD, Emery G. Evaluation of aid to diagnosis of pigmented skin lesions in general practice: controlled trial randomised by practice. BMJ. 2003;327(7411):375. PubMed PMID: 12919990. Pubmed Central PMCID: PMC175808. |  | | --- | --- | | Intervention does not include a diagnostic CPR as defined by review (uses CPR of Del Mar 1995 above) |
| | Forrest CB, Fiks AG, Bailey LC, Localio R, Grundmeier RW, Richards T, et al. Improving adherence to otitis media guidelines with clinical decision support and physician feedback. Pediatrics. 2013;131(4):e1071-81. PubMed PMID: 23478860. |  | | --- | --- | | Intervention does not include a diagnostic CPR |
| | Foy R, Penney GC, Grimshaw JM, Ramsay CR, Walker AE, MacLennan G, et al. A randomised controlled trial of a tailored multifaceted strategy to promote implementation of a clinical guideline on induced abortion care. BJOG. 2004 Jul;111(7):726-33. PubMed PMID: 15198764. |  | | --- | --- | | Intervention does not incorporate a diagnostic CPR |
| | Gonzales R, Anderer T, McCulloch CE, Maselli JH, Bloom FJ, Jr., Graf TR, et al. A cluster randomized trial of decision support strategies for reducing antibiotic use in acute bronchitis. JAMA Intern Med. 2013 Feb 25;173(4):267-73. PubMed PMID: 23319069. Pubmed Central PMCID: 3582762. |  | | --- | --- | | Intervention does not include a diagnostic CPR |
| | Hagiwara M, Henricson M, Jonsson A, Suserud BO. Decision-support tool in prehospital care: a systematic review of randomized trials. Prehospital & Disaster Medicine. 2011;26(5):319-29. PubMed PMID: 22030101. |  | | --- | --- | | Intervention does not include a diagnostic CPR |
| | Hess EP, Knoedler MA, Shah ND, Kline JA, Breslin M, Branda ME, et al. The chest pain choice decision aid: a randomized trial. Circulation Cardiovascular Quality & Outcomes. 2012;5(3):251-9. PubMed PMID: 22496116. |  | | --- | --- | | Intervention is a patient decision aid not a diagnostic CPR for clinician use only |
| | Hamilton E, Platt R, Gauthier R, McNamara H, Miner L, Rothenberg S, et al. The effect of computer-assisted evaluation of labor on cesarean rates. Journal for healthcare quality : official publication of the National Association for Healthcare Quality. 2004 Jan-Feb;26(1):37-44. PubMed PMID: 14763319. |  | | --- | --- | | Intervention does not include a diagnostic CPR as defined by review |
| | Hoffmann U, Truong QA, Fleg JL, Goehler A, Gazelle S, Wiviott S, et al. Design of the Rule Out Myocardial Ischemia/Infarction Using Computer Assisted Tomography: a multicenter randomized comparative effectiveness trial of cardiac computed tomography versus alternative triage strategies in patients with acute chest pain in the emergency department. American heart journal [Internet]. 2012; 163(3):[330-8, 8.e1 pp.]. |  | | --- | --- | | Intervention does not include a diagnostic CPR |
| | Kucher N, Koo S, Quiroz R, Cooper JM, Paterno MD, Soukonnikov B, et al. Electronic alerts to prevent venous thromboembolism among hospitalized patients. New England Journal of Medicine. 2005;352(10):969-77. PubMed PMID: 15758007. |  | | --- | --- | | Intervention does not include a diagnostic CPR developed in other data |
| | Kline JA, Zeitouni RA, Hernandez-Nino J, Jones AE. Randomized trial of computerized quantitative pretest probability in low-risk chest pain patients: effect on safety and resource use. Ann Emerg Med. 2009 Jun;53(6):727-35 e1. PubMed PMID: 19135281. |  | | --- | --- | | Study of a patient decision aid not a diagnostic CPR for clinician use only |
| Kline JA, Jones AE, Shapiro NI, Hernandez J, Hogg MM, Troyer J, et al. Multicenter, randomized trial of quantitative pretest probability to reduce unnecessary medical radiation exposure in emergency department patients with chest pain and dyspnea. Circulation Cardiovascular imaging. 2014 Jan;7(1):66-73. PubMed PMID: 24275953. | Study of a decision aid for patient and clinician use, not of a diagnostic CPR for clinician use only |
| | Lindley-Jones M, Finlayson BJ. Triage nurse requested x rays--are they worthwhile? J Accid Emerg Med. 2000 Mar;17(2):103-7. PubMed PMID: 10718230. Pubmed Central PMCID: 1725357. |  | | --- | --- | | Intervention does not include a diagnostic CPR |
| Llor C, Madurell J, Balague-Corbella M, Gomez M, Cots JM. Impact on antibiotic prescription of rapid antigen detection testing in acute pharyngitis in adults: a randomised clinical trial. Br J Gen Pract. 2011 May;61(586):e244-51. PubMed PMID: 21619748. Pubmed Central PMCID: 3080229. | Intervention does not include a diagnostic CPR |
| | Lewis G, Sharp D, Bartholomew J, Pelosi AJ. Computerized assessment of common mental disorders in primary care: effect on clinical outcome. Fam Pract. 1996 Apr;13(2):120-6. PubMed PMID: 8732321. |  | | --- | --- | | Intervention does not include a diagnostic CPR |
| | Lee NJ, Chen ES, Currie LM, Donovan M, Hall EK, Jia H, et al. The effect of a mobile clinical decision support system on the diagnosis of obesity and overweight in acute and primary care encounters. Advances in Nursing Science. 2009;32(3):211-21. PubMed PMID: 19707090. |  | | --- | --- | | Intervention does not include a diagnostic CPR |
| | Lironi A, Zawadynski S, La Scala G, Thevenod C, Le Coultre C. [Value of the Pediatric Trauma Score in routine hospital practice--apropos of a prospective one-year trial]. Swiss Surgery. 1999;5(6):271-5. PubMed PMID: 10608189. |  | | --- | --- | | Intervention does not include a diagnostic CPR |
| | Marrie TJ, Lau CY, Wheeler SL, Wong CJ, Vandervoort MK, Feagan BG. A controlled trial of a critical pathway for treatment of community-acquired pneumonia. CAPITAL Study Investigators. Community-Acquired Pneumonia Intervention Trial Assessing Levofloxacin. JAMA. 2000;283(6):749-55. PubMed PMID: 10683053. |  | | --- | --- | | Intervention does not include a diagnostic CPR |
| | Matic I, Titlic M, Dikanovic M, Jurjevic M, Jukic I, Tonkic A. Effects of APACHE II score on mechanical ventilation; prediction and outcome. Acta anaesthesiologica Belgica [Internet]. 2007; 58(3):[177-83 pp.]. |  | | --- | --- | | Intervention does not include a diagnostic CPR |
| | Moylan CA, Brady CW, Johnson JL, Smith AD, Tuttle-Newhall JE, Muir AJ. Disparities in liver transplantation before and after introduction of the MELD score. JAMA. 2008 Nov 26;300(20):2371-8. PubMed PMID: 19033587. Pubmed Central PMCID: 3640479. |  | | --- | --- | | Intervention does not include a diagnostic CPR |
| | Meyer G, Kopke S, Bender R, Muhlhauser I. Predicting the risk of falling--efficacy of a risk assessment tool compared to nurses' judgement: a cluster-randomised controlled trial [ISRCTN37794278]. BMC Geriatr. 2005;5:14. PubMed PMID: 16285880. Pubmed Central PMCID: 1312310. |  | | --- | --- | | Intervention does not include a diagnostic CPR |
| | McGregor JC, Weekes E, Forrest GN, Standiford HC, Perencevich EN, Furuno JP, et al. Impact of a computerized clinical decision support system on reducing inappropriate antimicrobial use: a randomized controlled trial. Journal of the American Medical Informatics Association. 2006;13(4):378-84. PubMed PMID: 16622162. Pubmed Central PMCID: PMC1513678. |  | | --- | --- | | Intervention does not include a diagnostic CPR |
| | Montgomery AA, Fahey T, Peters TJ, MacIntosh C, Sharp DJ. Evaluation of computer based clinical decision support system and risk chart for management of hypertension in primary care: randomised controlled trial. BMJ. 2000;320(7236):686-90. PubMed PMID: 10710578. Pubmed Central PMCID: PMC27312. |  | | --- | --- | | Intervention does not include a diagnostic CPR |
| | Montgomery AA, Emmett CL, Fahey T, Jones C, Ricketts I, Patel RR, et al. Two decision aids for mode of delivery among women with previous caesarean section: randomised controlled trial. BMJ (Clinical research ed) [Internet]. 2007; 334(7607):[1305 p.]. |  | | --- | --- | | Intervention does not include a diagnostic CPR |
| | Murray LS, Teasdale GM, Murray GD, Jennett B, Miller JD, Pickard JD, et al. Does prediction of outcome alter patient management? Lancet. 1993 Jun 12;341(8859):1487-91. PubMed PMID: 8099377. |  | | --- | --- | | Intervention does not include a diagnostic CPR |
| | Plank J, Blaha J, Cordingley J, Wilinska ME, Chassin LJ, Morgan C, et al. Multicentric, randomized, controlled trial to evaluate blood glucose control by the model predictive control algorithm versus routine glucose management protocols in intensive care unit patients. Diabetes care [Internet]. 2006; 29(2):[271-6 pp.]. |  | | --- | --- | | Intervention does not include a diagnostic CPR |
| | Paul M, Andreassen S, Tacconelli E, Nielsen AD, Almanasreh N, Frank U, et al. Improving empirical antibiotic treatment using TREAT, a computerized decision support system: cluster randomized trial. J Antimicrob Chemother. 2006 Dec;58(6):1238-45. PubMed PMID: 16998208. |  | | --- | --- | | Intervention does not include a diagnostic CPR |
| | Protheroe J, Bower P, Chew-Graham C, Peters TJ, Fahey T. Effectiveness of a computerized decision aid in primary care on decision making and quality of life in menorrhagia: results of the MENTIP randomized controlled trial. Medical decision making : an international journal of the Society for Medical Decision Making [Internet]. 2007; 27(5):[575-84 pp.]. |  | | --- | --- | | Intervention does not include a diagnostic CPR |
| | Radecki SE, Brunton SA. Randomized clinical trial of a diagnostic instrument for pain complaints. Family medicine [Internet]. 1999; 31(10):[713-21 pp.]. |  | | --- | --- | | Intervention does not include a diagnostic CPR |
| | Ross MA, Compton S, Medado P, Fitzgerald M, Kilanowski P, O'Neil BJ. An emergency department diagnostic protocol for patients with transient ischemic attack: a randomized controlled trial. Annals of emergency medicine [Internet]. 2007; 50(2):[109-19 pp.]. |  | | --- | --- | | Intervention does not include a diagnostic CPR |
| | Ross MA, Kilanowski P, Mattke A, B ON, Compton S. The Emergency Department Transient Ischemic Attack Accelerated Diagnostic Protocol (TIA ADP) Study. Annals of Emergency Medicine [Internet]. 2004; 44(4 Suppl 1):[S121 p.]. |  | | --- | --- | | Intervention does not include a diagnostic CPR |
| | Schriger DL, Gibbons PS, Langone CA, Lee S, Altshuler LL. Enabling the diagnosis of occult psychiatric illness in the emergency department: a randomized, controlled trial of the computerized, self-administered PRIME-MD diagnostic system. Ann Emerg Med. 2001 Feb;37(2):132-40. PubMed PMID: 11174229. |  | | --- | --- | | Intervention does not include a diagnostic CPR as defined by review |
| | Thomas RE, Croal BL, Ramsay C, Eccles M, Grimshaw J. Effect of enhanced feedback and brief educational reminder messages on laboratory test requesting in primary care: a cluster randomised trial. Lancet. 2006 Jun 17;367(9527):1990-6. PubMed PMID: 16782489. |  | | --- | --- | | Intervention does not include a diagnostic CPR |
| | Visser FJ, van der Vegt MJ, van der Wilt GJ, Janssen JP. The optimization of the diagnostic work-up in patients with suspected obstructive lung disease. BMC Pulmonary Medicine. 2010;10:60. PubMed PMID: 21092293. Pubmed Central PMCID: PMC2996350. |  | | --- | --- | | Intervention does not include a diagnostic CPR |
| | van Wijk MA, van der Lei J, Mosseveld M, Bohnen AM, van Bemmel JH. Assessment of decision support for blood test ordering in primary care. a randomized trial. Ann Intern Med. 2001 Feb 20;134(4):274-81. PubMed PMID: 11182837. |  | | --- | --- | | Intervention does not include a diagnostic CPR |
| Study is a systematic review of ‘decision support tools’ (these were checked for relevant trials) | |
| | Bright TJ, Wong A, Dhurjati R, Bristow E, Bastian L, Coeytaux RR, et al. Effect of clinical decision-support systems: a systematic review. Annals of Internal Medicine. 2012;157(1):29-43. PubMed PMID: 22751758. |  | | --- | --- | | References checked |
| | Cleveringa FG, Gorter KJ, van den Donk M, van Gijsel J, Rutten GE. Computerized decision support systems in primary care for type 2 diabetes patients only improve patients' outcomes when combined with feedback on performance and case management: a systematic review. Diabetes Technology & Therapeutics. 2013;15(2):180-92. PubMed PMID: 23360424. |  | | --- | --- | | References checked |
| | Fillmore CL, Bray BE, Kawamoto K. Systematic review of clinical decision support interventions with potential for inpatient cost reduction. BMC Med Inform Decis Mak. 2013;13:135. PubMed PMID: 24344752. Pubmed Central PMCID: 3878492. |  | | --- | --- | | References checked |
| | Garg AX, Adhikari NK, McDonald H, Rosas-Arellano MP, Devereaux PJ, Beyene J, et al. Effects of computerized clinical decision support systems on practitioner performance and patient outcomes: a systematic review. JAMA. 2005;293(10):1223-38. PubMed PMID: 15755945. |  | | --- | --- | | References checked |
| | Hagiwara M, Henricson M, Jonsson A, Suserud BO. Decision-support tool in prehospital care: a systematic review of randomized trials. Prehospital & Disaster Medicine. 2011;26(5):319-29. PubMed PMID: 22030101. |  | | --- | --- | | References checked |
| | Roshanov PS, Misra S, Gerstein HC, Garg AX, Sebaldt RJ, Mackay JA, et al. Computerized clinical decision support systems for chronic disease management: a decision-maker-researcher partnership systematic review. Implementation Science. 2011;6:92. PubMed PMID: 21824386. Pubmed Central PMCID: PMC3170626. |  | | --- | --- | | References checked |
| | Roshanov PS, You JJ, Dhaliwal J, Koff D, Mackay JA, Weise-Kelly L, et al. Can computerized clinical decision support systems improve practitioners' diagnostic test ordering behavior? A decision-maker-researcher partnership systematic review. Implementation Science. 2011;6:88. PubMed PMID: 21824382. Pubmed Central PMCID: PMC3174115. |  | | --- | --- | | References checked |
| | Stengel D, Bauwens K, Rademacher G, Ekkernkamp A, Guthoff C. Emergency ultrasound-based algorithms for diagnosing blunt abdominal trauma. The Cochrane database of systematic reviews. 2013;7:CD004446. PubMed PMID: 23904141. |  | | --- | --- | | References checked |
| Wang, R. C., Bent, S., Weber, E., Neilson, J., Smith-Bindman, R., & Fahimi, J. The Impact of Clinical Decision Rules on Computed Tomography Use and Yield for Pulmonary Embolism: A Systematic Review and Meta-analysis. Annals of Emergency Medicine*.* 2016; 67(6):693-701 | References checked |
| **Protocol or in-progress impact study** | |
| Chew DP, Astley CM, Luker H, Alprandi-Costa B, Hillis G, Chow CK, et al. A cluster randomized trial of objective risk assessment versus standard care for acute coronary syndromes: Rationale and design of the Australian GRACE Risk score Intervention Study (AGRIS). American Heart Journal. 2015;170(5):995-1004.e1. PubMed PMID: 26542510. | Protocol for an in progress study |
| Freund Y, Rousseau A, Guyot-Rousseau F, Claessens YE, Hugli O, Sanchez O, et al. PERC rule to exclude the diagnosis of pulmonary embolism in emergency low-risk patients: study protocol for the PROPER randomized controlled study. Trials [Electronic Resource]. 2015;16:537. PubMed PMID: 26607669. | Protocol for an in progress study |
| | Hess EP, Wyatt KD, Kharbanda AB, Louie JP, Dayan PS, Tzimenatos L, et al. Effectiveness of the head CT choice decision aid in parents of children with minor head trauma: study protocol for a multicenter randomized trial. Trials. 2014;15:253. PubMed PMID: 24965659. Pubmed Central PMCID: 4081461. |  | | --- | --- | | Protocol for an in progress study |
| | Murray GD. Assessing the clinical impact of a predictive system in severe head injury. Medical informatics = Medecine et informatique. 1990 Jul-Sep;15(3):269-73. PubMed PMID: 2232962. |  | | --- | --- | | Protocol |
| | Mann DM, Kannry JL, Edonyabo D, Li AC, Arciniega J, Stulman J, et al. Rationale, design, and implementation protocol of an electronic health record integrated clinical prediction rule (iCPR) randomized trial in primary care. Implementation science : IS [Internet]. 2011; 6:[109 p.]. |  | | --- | --- | | Protocol for study of McGinn (included in review) |
| | Pierce MA, Hess EP, Kline JA, Shah ND, Breslin M, Branda ME, et al. The Chest Pain Choice trial: a pilot randomized trial of a decision aid for patients with chest pain in the emergency department. Trials. 2010;11:57. PubMed PMID: 20478056. Pubmed Central PMCID: 2881067. |  | | --- | --- | | Protocol for the in progress study of Hess ( in this section above) |
| | Poldervaart JM, Reitsma JB, Koffijberg H, Backus BE, Six AJ, Doevendans PA, et al. The impact of the HEART risk score in the early assessment of patients with acute chest pain: design of a stepped wedge, cluster randomised trial. BMC Cardiovasc Disord. 2013;13:77. PubMed PMID: 24070098. Pubmed Central PMCID: 3849098. |  | | --- | --- | | Submitted to journal for publication (advice from investigator) |
| | Stiell IG, Grimshaw J, Wells GA, Coyle D, Lesiuk HJ, Rowe BH, et al. A matched-pair cluster design study protocol to evaluate implementation of the Canadian C-spine rule in hospital emergency departments: Phase III. Implement Sci. 2007;2:4. PubMed PMID: 17288613. Pubmed Central PMCID: 1802999. |  | | --- | --- | | Protocol for study of Stiell 2009 (included in review) |
| Other | |
| | Chusak O. Prediction of Late-Onset Neonatal Sepsis Using LNS Score Comparing with Physicians' Probability Estimates: A Cluster Randomized Trial. Pediatric Academic Society [Internet]. 2008; http://www.abstracts2view.com/pas/(469). |  | | --- | --- | | Unable to obtain full text |
| | Corey GA, Merenstein JH. Applying the acute ischemic heart disease predictive instrument. The Journal of family practice [Internet]. 1987; 25(2):[127-33 pp.]. |  | | --- | --- | | Reference standard not current |
| | Kurashima S, Kobayashi K, Toyabe S, Akazawa K. Accuracy and efficiency of computer-aided nursing diagnosis. International journal of nursing terminologies and classifications : the official journal of NANDA International [Internet]. 2008; 19(3):[95-101 pp.]. |  | | --- | --- | | Study not in real patients |
| | Murray GD, Murray LS, Barlow P, Teasdale GM, Jennett WB. Assessing the performance and clinical impact of a computerized prognostic system in severe head injury. Stat Med. 1986 Sep-Oct;5(5):403-10. PubMed PMID: 3538261. |  | | --- | --- | | Comparison in paper cases |
| | Tierney WM, McDonald CJ, Hui SL, Martin DK. Computer predictions of abnormal test results. Effects on outpatient testing. JAMA. 1988 Feb 26;259(8):1194-8. PubMed PMID: 3339821. |  | | --- | --- | | CPR not for clinical diagnosis |
| | Wexler JR, Swender PT, Tunnessen WW, Jr., Oski FA. Impact of a system of computer-assisted diagnosis. Initial evaluation of the hospitalized patient. Am J Dis Child. 1975 Feb;129(2):203-5. PubMed PMID: 1091140. |  | | --- | --- | | CPR for diagnosis across multiple body systems |
