# Supplementary material for: Systematic review of the effects of care provided with and without diagnostic clinical prediction rules
Source: Diagn Progn Res. 2017 Apr 26;1:13. doi: 10.1186/s41512-017-0013-2 (PMC6460683; doi:10.1186/s41512-017-0013-2)
Supplement: Supplementary file 4 — Primary and secondary outcomes reported in the included studies. (DOC 61 kb) [file 41512_2017_13_MOESM4_ESM.doc]

**Additional File 4**

Primary and secondary outcomes reported in the included studies

|  | Patient outcomes | Process of care | Clinical Decisions | Accuracy | Use and application |
| --- | --- | --- | --- | --- | --- |
| Worrall et al 2007 |  |  | X |  |  |
| McIsaac & Goel 1998 |  |  | X |  |  |
| McIsaac et al 2002 |  |  | X |  |  |
| McGinn et al 2013 |  |  | X |  | x |
| Little et al 2013 | X |  | x |  |  |
| Douglas et al 2000 |  | X | x |  |  |
| Farahnak et al 2007 |  | X | x |  |  |
| Lintula et al 2010 |  |  |  | X |  |
| Lintula et al 2009 |  |  |  | X |  |
| Wellwood et al 1992 | x |  | x | X |  |
| Roukema et al 2008 |  | X |  |  |  |
| Lacroix et al 2014 |  | x | X |  |  |
| de Vos-Kerkhof et al 2015 |  | x | X |  |  |
| Auleley et al 1997 | x |  | X |  | x |
| Fan et al 2006 | x | X | x |  |  |
| Than et al 2014 | x |  | X |  |  |
| Mahler et al 2015 |  | x | X |  |  |
| Sanchis et al 2010 |  | X |  |  |  |
| Torres et al 2014 | x |  | X |  |  |
| Ferrero et al, 2015 | x |  | X |  |  |
| Klassen et al 1993 |  | x | X |  |  |
| Walter et al 2012 |  | x | X | x |  |
| Rodger et al 2006 | X |  | x |  |  |
| Horowitz et al 2007 | X | x |  |  |  |
| Bogusevicius et al 2002 |  | x |  | X |  |
| Stiell et al 2010 |  |  | X | x | x |
| Stiell et al 2009 |  |  | X |  | x |
