# Supplementary material for: Systematic review of the effects of care provided with and without diagnostic clinical prediction rules
Source: Diagn Progn Res. 2017 Apr 26;1:13. doi: 10.1186/s41512-017-0013-2 (PMC6460683; doi:10.1186/s41512-017-0013-2)
Supplement: Supplementary file 5 — Risk of bias graph. Review authors’ judgments about each risk of bias domain for each included study. (DOC 190 kb) [file 41512_2017_13_MOESM5_ESM.doc]

**Additional File 5**

Review authors’ judgments about risk of bias in the included studies

| **Domain of bias** | **Selection bias** | | | **Performance bias** | | **Detection bias** | **Attrition bias** | **Reporting bias** | **Other bias** | | | |
| --- | --- | --- | --- | --- | --- | --- | --- | --- | --- | --- | --- | --- |
| Specific criteria | **Random sequence generation** | **Allocation concealment** | | **Blinding of participants/ personnel** | | **Blinding of outcome assessment** | **Incomplete outcome data** | **Selective reporting** | **Recruitment bias *** | **Baseline imbalance*** | **Incorrect analysis*** | **Contamination** |
| **Sore throat** | | | | | | | | | | | | |
| Worrall et al., 2007 | Low | Unclear | | High | | Unclear | High | Unclear | Unclear | Unclear | High | Low |
| McIsaac & Goel 1998 | Unclear | Unclear | | Unclear | | Unclear | High | Unclear | - | - | - | Low |
| McIsaac et al., 2002 [2] | Unclear | Unclear | | High | | Unclear | High | Unclear | High | Low | Low | Low |
| McGinn et al., 2013† | Low | Unclear | | High | | Unclear | Low | Low | Low | Low | Low | Unclear |
| Little et al., 2013 [1] | Low | Low | | High | | Unclear | Low | Low | - | - | - | High |
| **Acute appendicitis** | | | | | | | | | | | | |
| Douglas et al., 2000 [8] | Low | High | | High | | Unclear | Low | Unclear | - | - | - | Low |
| Farahnak et al., 2007 [9] | Low | Unclear | | High | | Unclear | Low | Unclear | - | - | - | Low |
| Lintula et al., 2010 [11] | Unclear | Unclear | | High | | Unclear | Low | Unclear | - | - | - | High |
| Lintula et al., 2009 [10] | Unclear | Low | | High | | Unclear | Low | Unclear | - | - | - | High |
| Wellwood et al., 1992 [6] | Low | Unclear | | High | | Unclear | Low | Unclear | Low | Unclear | Low | Unclear |
| **Serious infection in children with fever** | | | | | | | | | | | | |
| Roukema et al., 2008 [12] | Low | | Unclear | | High | Low | High | Unclear | - | - | - | Low |
| Lacroix et al., 2014 [14] | Low | | Unclear | | High | Unclear | Low | Low |  |  |  | Low |
| deVos-Kerkhof et al., 2015 [13] | Unclear | | Unclear | | High | Unclear | Low | Unclear | - | - | - | Low |
| **Possible cardiac chest pain** | | | | | | | | | | | | |
| Than et al., 2014 [16] | Low | | Low | | High | Low | Low | Low | - | - | - | Low |
| Mahler et al., 2015 [17] | Low | | Low | | High | Low | Low | Low |  |  |  | High |
| Sanchis et al., 2010 [15] | Low | | Low | | High | Unclear | Low | Low | - | - | - | Low |
| **Ankle or mid-foot injury** | | | | | | | | | | | | |
| Auleley et al., 1997 [20] | Unclear | | Unclear | | High | Unclear | Low | Unclear | Low | Unclear | Low | Low |
| Fan et al., 2006 [21] | Unclear | | Low | | Low | Low | High | Unclear | - | - | - | Low |
| **Bacterial pneumonia** | | | | | | | | | | | | |
| Ferrero et al., 2015 [18] | Unclear | | High | | High | Unclear | Low | Unclear |  |  |  | High |
| Torres et al., 2014 [19] | Unclear | | Unclear | | High | Low | Low | Unclear | - | - | - | Low |
| McGinn et al., 2013† [5] | Low | | Unclear | | High | Unclear | Low | Low | Low | Low | Low | Unclear |
| **Single studies of different clinical conditions** | | | | | | | | | | | | |
| Klassen et al., 1993 [22] | Low | | Unclear | | Low | Low | Low | Unclear | - | - | - | Low |
| Walter et al., 2012 [23] | Low | | Low | | High | High | Low | Low | - | - | - | Low |
| Rodger et al., 2006 [24] | Unclear | | Low | | Low | Low | Low | Unclear | - | - | - | Low |
| Horowitz et al., 2007 [25] | Unclear | | Unclear | | High | High | Low | Unclear | Unclear | Unclear | High | Low |
| Bogusevicius et al., 2002 [26] | Unclear | | Low | | High | Unclear | Low | Unclear | - | - | - | Low |
| Stiell et al., 2010 [27] | Unclear | | Unclear | | High | Unclear | Low | Unclear | Low | Low | Low | Low |
| Stiell et al., 2009 [28] | Low | | Unclear | | High | Unclear | Low | Low | Low | Low | Low | Low |

*For trials randomising centres or clinics or individual clinicians who then recruit participants to the study; †this study evaluated two prediction rules for different clinical conditions
