# Supplementary material for: Systematic review of the effects of care provided with and without diagnostic clinical prediction rules
Source: Diagn Progn Res. 2017 Apr 26;1:13. doi: 10.1186/s41512-017-0013-2 (PMC6460683; doi:10.1186/s41512-017-0013-2)
Supplement: Supplementary file 6 — The performance of included studies against the minimum required elements for reporting of diagnostic strategies and implementation methods. (DOC 242 kb) [file 41512_2017_13_MOESM6_ESM.doc]

**Additional File 6**

The performance of included studies against the minimum required elements for reporting of diagnostic strategies and implementation methods

|  | **Intervention description** | | | | | **Implementation description** | |
| --- | --- | --- | --- | --- | --- | --- | --- |
| **Study** | **Primary outcome** | **Study arm** | **Diagnostic tests being evaluated are described?** | **Criteria for arriving at a diagnosis, testing or treatment decision are described?** | **Selection of treatment and how it is administered is described?** | **Training of clinicians in use of the CPR or application of its output is provided and if provided, is described?** | **Method by which the CPR is made available for use is described?** |
| **Group A streptococcus throat infection** | | | | | | | |
| **Worrall et al., 2007** | Antibiotic prescribing | Experimental | Yes | Yes | NA | No | No |
|  |  | Control | No | No |  |  |  |
| **McIsaac & Goel 1998** | Antibiotic prescribing | Experimental | Yes | Yes | NA | No | Yes |
|  |  | Control | Yes | No |  |  |  |
| **McIsaac et al., 2002** | Unnecessary antibiotic prescribing | Experimental | Yes | Yes | NA | No | Yes |
|  |  | Control | Yes | No |  |  |  |
| **McGinn et al., 2013** | Antibiotic prescribing | Experimental | Yes | Yes | NA | Yes | Yes |
|  |  | Control | No | No |  |  |  |
| **Little et al., 2013** | Symptom severity | Experimental | Yes | Yes | No | No | No |
|  |  | Control | Yes | No | No |  |  |
| **Acute appendicitis** | | | | | |  |  |
| **Douglas et al., 2000** | Time to therapeutic operation | Experimental | Yes | Yes | NA | No | No |
|  |  | Control | No | No |  |  |  |
| **Farahnak et al., 2007** | Time to surgery | Experimental | Yes | Yes | NA | No | No |
|  |  | Control | No | No |  |  |  |
| **Lintula et al., 2010** | Diagnostic accuracy | Experimental | Yes | Yes | NA | Yes | No |
|  |  | Control | Yes | No |  |  |  |
| **Lintula et al., 2009** | Diagnostic accuracy | Experimental | Yes | Yes | NA | Yes | No |
|  |  | Control | Yes | No |  |  |  |
| **Wellwood et al., 1992** | Diagnostic accuracy | Experimental | No | No | NA | No | Yes |
|  |  | Control | No | No |  |  |  |
| **Serious bacterial infection in children with fever** | | | | | | | |
| **Roukema et al., 2008** | Length of stay | Experimental | Yes | Yes | NA | Yes | Yes |
|  |  | Control | No | No |  |  |  |
| **Lacroix et al., 2014** | Antibiotic prescribing | Experimental | Yes | Yes | NA | Yes | No |
|  |  | Control | Yes | No |  |  |  |
| **de Vos-Kerkhof et al., 2015** | Appropriate test use | Experimental | Yes | Yes | NA | Yes | Yes |
|  |  | Control | No | No |  |  |  |

|  | **Intervention description** | | | | | **Implementation description** | |
| --- | --- | --- | --- | --- | --- | --- | --- |
| **Study** | **Primary outcome** | **Study arm** | **Diagnostic tests being evaluated are described?** | **Criteria for arriving at a diagnosis, testing or treatment decision are described?** | **Selection of treatment and how it is administered is described?** | **Training of clinicians in use of the CPR or application of its output is provided and if provided, is described?** | **Method by which the CPR is made available for use is described?** |
| **Ankle or mid-foot fracture** | | | | | |  |  |
| **Auleley et al., 1997** | Radiography requests | Experimental | No | No | NA | Yes | Yes |
|  |  | Control | No | No |  |  |  |
| **Fan et al., 2006** | Length of stay in the emergency department | Experimental | No | No | NA | Yes | Yes |
|  |  | Control | No | No |  |  |  |
| **Acute coronary syndromes** | | | | | |  |  |
| **Than et al., 2014** | Safe discharge | Experimental | Yes | Yes | NA | No | No |
|  |  | Control | Yes | No |  |  |  |
| **Mahler et al., 2015** | Objective cardiac testing | Experimental | Yes | Yes | NA | No | Yes |
|  |  | Control | Yes | No |  |  |  |
| **Sanchis et al., 2010** | Hospitalisation | Experimental | Yes | Yes | NA | No | No |
|  |  | Control | Yes | Yes |  |  |  |
| **Bacterial pnuemonia** | | | | | |  |  |
| **Torres et al., 2014** | Antibiotic prescribing | Experimental | Yes | Yes | NA | No | No |
|  |  | Control | Yes | No |  |  |  |
| **Ferrero et al., 2015** | Antibiotic prescribing | Experimental | Yes | Yes | NA | No | No |
|  |  | Control | Yes | No |  |  |  |
| **Single studies of different clinical conditions** | | | | | | | |
| **McGinn et al., 2013** | Antibiotic prescribing | Experimental | Yes | Yes | NA | Yes | Yes |
|  |  | Control | No | No |  |  |  |
| **Klassen et al., 1993** | Radiography requests | Experimental | Yes | Yes | NA | Yes | No |
|  |  | Control | No | No |  |  |  |
| **Walter et al., 2012** | Appropriate referral | Experimental | Yes | Yes | NA | Yes | Yes |
|  |  | Control | Yes | No |  |  |  |
| **Rodger et al., 2006** | Venous thromboembolic events | Experimental | Yes | Yes | No | No | No |
|  |  | Control | No | No | No |  |  |
| **Horowitz et al., 2007** | Symptom relief | Experimental | Yes | Yes | No | No | No |
|  |  | Control | No | No | No |  |  |
| **Bogusevicius et al., 2002** | Diagnostic accuracy | Experimental | Yes | No | NA | No | Yes |
|  |  | Control | Yes | Yes |  |  |  |
| **Stiell et al., 2010** | Computed tomography scan rates | Experimental | Yes | Yes | NA | Yes | No |
|  |  | Control | No | No |  |  |  |
| **Stiell et al., 2009** | Cervical spine imaging rates | Experimental | Yes | Yes | NA | Yes | No |
|  |  | Control | No | No |  |  |  |
