# Supplementary material for: Systematic review of the effects of care provided with and without diagnostic clinical prediction rules
Source: Diagn Progn Res. 2017 Apr 26;1:13. doi: 10.1186/s41512-017-0013-2 (PMC6460683; doi:10.1186/s41512-017-0013-2)
Supplement: Supplementary file 7 — Results of studies of diagnostic CPRs for suspected Group A Streptococcus throat infection by outcome. Results of studies of diagnostic CPRs for suspected acute appendicitis by outcome. Results of studies of diagnostic CPRs for suspected serious bacterial infection in children with fever. Results of studies of diagnostic CPRs for suspected acute coronary syndrome by outcome. Results of studies of diagnostic CPRs for suspected (bacterial) pneumonia by outcome. Results of studies of diagnostic CPRs for suspected ankle or mid-foot fracture by outcome. Results of single studies of diagnostic CPRs for different clinical conditions. (DOC 538 kb) [file 41512_2017_13_MOESM7_ESM.doc]

**Additional File 7**

Results of studies of diagnostic CPRS for suspected Group A Streptococcus throat infection by outcome

| Outcomes | Number of trials/ N of trial or comparison | Result | |
| --- | --- | --- | --- |
| Patient outcomes | | | |
| Symptom severity (6 point scale)  Resolution of symptoms rated moderately bad or worse  Return within 1 month with sore throat  Suppurative complications | 1 (Little et al., )*/336  1 (Little et al., )*/334 | CPR only MD^†^ -0.33 (95% CI -0.64 to -0.02)  CPR + RADT MD^†^ -0.30 (95% CI -0.61 to 0.00)  CPR only HR^†^ 1.30 (95% CI 1.03 to 1.63)  CPR + RADT HR^†^ 1.11 (95% CI 0.88 to 1.40)  CPR only RR^‡^ 0.91 (95% CI 0.47 to 1.72)  CPR + RADT RR^‡^ 0.74 (95% CI 0.36 to 1.47)  no suppurative complications during the trial | |
| Process outcomes | | | |
| Proportion of visits resulting in return to ED 2 weeks after initial visit | 1 (McGinn et al., 2013 [5]/984‡ | 0.7% | 0.5% p>0.9 |
| Proportion of visits resulting in outpatient clinic visits 2 weeks after initial visit | 1 (McGinn et al., 2013 [5]/984‡ | 7.7% | 11.3% p=0.1 |
| Clinical decisions/appropriateness of clinical decisions | | | |
| Antibiotic prescriptions  Unnecessary antibiotic prescriptions^§^  Antibiotic prescription by CPR recommendation  No swab, no script  Swab, no script  Script if early or swab  Throat swab by CPR recommendation  No swab, no script  Swab, no script  Script if early or swab  Rapid streptococcal test orders  Pharyngitis throat culture orders | 5 (Little et al., , McIsaac et al., , Worrall et al., *, McIsaac & Goel *, McGinn et al., *)  1 (Little et al., )/334  1 (Worrall et al.,)/243  1 (McIsaac et al.,*)/621  1 (McIsaac & Goel )/396  1 (McIsaac & Goel)/396  1 (McGinn et al.,)/598  1 (McGinn et al.,)/598 | CPR only pooled RR 0.86 (95%CI 0.75 to 0.99)  CPR + RADT RR^†^ 0.73 (95%CI 0.52 to 0.98)  CPR + RADT RR 0.66 (95%CI 0.5 0 to 0.87)  OR 0.76^ǁ^ (95%CI 0.42 to 1.40)  OR 0.30 (95%CI 0.05 to 1.18)  OR 0.55 (95%CI 0.26 to 1.15)  OR 0.71 (95%CI 0.01 to 59.77)  OR 0.80 (95%CI 0.44 to 1.44)  OR 2.44 (95%CI 0.84 to 7.67)  OR 1.73 (95%CI 0.289 to 12.70)  RR^¶^ 0.75 (95%CI 0.58 to 0.97)  RR^¶^ 0.54 (95%CI 0.18 to 1.64) | |
| Use and application outcomes^**^ | | | |
| Decision support tool opened  CPR calculator opened | 1 (McGinn et al.,)/598 | 74.3%  66.6% | |

CPR – clinical prediction rule; MD – mean difference; RADT – rapid antigen detection test; HR – hazard ratio; OR – odds ratio; RR – risk ratio

*primary outcome of the trial; †adjusted for baseline severity and fever; ‡Adjusted for baseline severity, fever and previous antibiotic use; ^§^prescription of an antibiotic when throat culture negative; ^ǁ^ Adjusted for patient and physician characteristics; ^¶^Adjusted for age; **only relevant to the intervention arm of studies where use of the CPR was discretionary

Results of studies of diagnostic CPRs for suspected acute appendicitis by outcome

| Outcomes | Number of trials/N of trial or comparison | Result | |
| --- | --- | --- | --- |
|  |  | **Intervention** | **Control** |
| Patient outcomes | | | |
| Perforated appendix rate | 1 (Wellwood et al., *)/4194 | CPR vs judgment with no diagnostic aid RR 0.47 (95%CI 0.19 to 1.15)  CPR vs judgment with SDC form RR 0.81 (95%CI 0.31 to 2.16) | |
| Process outcomes | | | |
| Time to therapeutic operation (mean hrs) | 1 (Douglas et al., †)/302 | 7.0 (95% CI 5.9 to 8.1) | 10.2 (95%CI 7.9 to 13) p=0.01 |
| Time to surgery  (median hrs) | 1 (Farahnak et al., †)/42 | 2.05 | 8.35 p=0.03^†^ |
| Duration of hospitalisation  (mean hrs)  Duration of hospitalisation  (median hrs) | 1 (Douglas et al., )/302  1 (Farahnak et al., )/42 | 53.4 (95% CI 47 to 60)  37.00 | 54.5 (95%CI 46 to 63) p=0.84^‡^  60.40 p=0.03^‡^ |
| Clinical decisions/appropriateness of clinical decisions | | | |
| Nontherapeutic operations | 5 /931 | Pooled RR 0.68 (95%CI 0.43 to 1.08) | |
| Admissions | 2 (Farahnak et al., )/42  (Wellwood et al., )/2596  (Wellwood et al., )/2584 | RR 0.72 (95%CI 0.49 to 1.05)  CPR vs judgment with no diagnostic aid RR 0.90 (95%CI 0.82 to 0.99)  CPR vs judgment with SDC form RR 1.0 (95%CI 0.91 to 1.12) | |
| Delayed treatment in association with perforation§ | 2 (Farahnak et al., )/42  (Douglas et al., )/302 | RR 3.0 (95%CI 0.13 to 69.7)  RR 2.22 (95%CI 0.44 to 11.26) | |
| Accuracy | | | |
| Sensitivity of initial examination | 3 (Lintula et al.,†)/126  (Lintula et al.,†)/177  (Wellwood et al.,†)/2596  (Wellwood et al.,)/2584 | 83%  87%  48% | 85% p=NS^‡^  89% p=NS^‡^  28% judgment with no diagnostic aid p=0.01^‡^  42% judgment with SDC form p=0.48^‡^ |
| Specificity of initial examination | 3 (Lintula et al., )/126  (Lintula et al., )/177  (Wellwood et al., )/2596  (Wellwood et al., )/2584 | 69%  59%  98% | 52% p=NS^‡^  80% p=0.03^‡^  96% judgment with no diagnostic aid p=0.04^‡^  96% judgment with SDC form P=0.01^†^ |
| Sensitivity of final examination ^ǁ^ | 2 (Lintula et al., )/126  (Lintula et al., )/177 | 100%  87% | 96% p=NS^‡^  100% p=0.02^‡^ |
| Specificity of final examination ^ǁ^ | 2 (Lintula et al., )/126  (Lintula et al., )/177 | 88%  98% | 67% p=0.03^‡^  84% p=0.03^‡^ |

CPR – clinical prediction rule; NS – not significant; SDC – structured data collection

*Data from this outcome are from the original study report and systematic review by the same author; †primary outcome;‡p value for difference between intervention and control groups; §patients with perforation where surgery not started within 10 hours of randomisation; ǁ>3 hours after initial examination

Results of studies of diagnostic CPRs for suspected serious bacterial infection in children with fever

| Outcomes | Number of trials/N of trial or comparison | Result | |
| --- | --- | --- | --- |
|  |  | **Intervention** | **Control** |
| Process outcomes | | | |
| Length of stay in the ED (median minutes (25^th^ to 75^th^ percentile)) | 2 (Roukema et al., 2008 )*/164  (de Vos-Kerkhof et al., 2015 )/439 | 138 (104 to 181)  117 (84 to 158) | 123 (83 to 179)(p=0.16)†  114 (81 to 162)(p>0.05)† |
| Clinical decisions/appropriateness of clinical decisions | | | |
| Antibiotic prescriptions | 1 (Lacroix et al., 2014 )*/271 | Clinician + CPR 41%  CPR alone 31% | 42% (p=0.88)  42% (p=0.009) in entire study cohort |
| Laboratory tests ordered when recommended by CPR (%) | 1 (Roukema et al., 2008 )/164 | 82%‡ | 44% (p<0.002)† |
| Chest x-ray performed when no pneumonia (false positive) | 1 (de Vos-Kerkhof et al., 2015 )*/439 | 60% | 57% (p>0.05) |
| No chest x-ray performed when pneumonia (false negative) | 1 (de Vos-Kerkhof et al., 2015 )*/439 | 1% | 1% (p>0.05) |
| Urine culture performed when no UTI (false positive) | 1 (de Vos-Kerkhof et al., 2015 )*/439 | 67% | 53% (p>0.05) |
| No urine culture performed when UTI (false negative) | 1 (de Vos-Kerkhof et al., 2015 )*/439 | 0% | 0.5% (p>0.05) |
| Urine dipstick performed | 1 (de Vos-Kerkhof et al., 2015 )/439 | 71% | 61% (p>0.05) |
| Overall diagnostics (minus urine dipstick analysis) | 1 (de Vos-Kerkhof et al., 2015 )/439 | 57% | 63% (p>0.05) |
| Antibiotics prescribed at discharge | 1 (de Vos-Kerkhof et al., 2015 )/439 | 32% | 36% (p>0.05) |
| % with SBI prescribed antibiotics | 1 (de Vos-Kerkhof et al., 2015 )/439 | 93% | 93% (p>0.05) |
| % with no SBI prescribed antibiotics | 1 (de Vos-Kerkhof et al., 2015 )/439 | 23% | 27% (p>0.05) |
| Hospitalisation | 2 (de Vos-Kerkhof et al., 2015 )/439  (Lacroix et al., 2014 )/271 | 12%  34% | 11% (p>0.05)  36% (p=0.81) |
| Accuracy | | | |
| Area under the curve for any SBI | 1 (Lacroix et al., 2014 )/271 | 0.91 (95%CI 0.87 to 0.95) | |
| Area under the curve for pneumonia | 1 (de Vos-Kerkhof et al., 2015 )/439 | 0.83 (95%CI 0.75 to 0.90) | |
| Area under the curve for other SBI | 1 (de Vos-Kerkhof et al., 2015 )/439 | 0.81 (95%CI 0.72 to 0.90) | |

CPR – clinical prediction rule; SBI – serious bacterial infection; UTI – urinary tract infection

*primary outcome; †p value for difference between intervention and control groups; ‡adjusted for age

Results of studies of diagnostic CPRS for suspected acute coronary syndrome by outcome

| Outcomes | Number of trials/N of trial or comparison | Result | |
| --- | --- | --- | --- |
|  |  | **Intervention** | **Control** |
| Patient outcomes | | | |
| Death or myocardial infarction at 1 year follow-up | 1 (Sanchis et al., 2009 )/320 | HR 1.9 (95%CI 0.7 to 5.2) | |
| Major adverse cardiac event at 30 day follow-up for patients discharged within 6 hours | 1 (Than et al., 2014)/542 | 1 | 0 |
| Major adverse cardiac event at 30 day follow-up for patients discharged without objective cardiac testing | 1 (Mahler et al., 2015 )/282 | 0 | 0 |
| Process outcomes | | | |
| Length of stay (median hrs) | 1 (Mahler et al., 2015 )/282 | 9.9 | 21.9 p=0.01 |
| Clinical decisions/appropriateness of clinical decisions | | | |
| Discharge within 6 hours without major adverse cardiac event within 30 days | 1 (Than et al., 2014 *)/542 | OR 1.9 (95%CI 1.2 to 3.1) | |
| Hospitalisation at index episode | 1 (Sanchis et al.,2009 *)/320 | OR 0.6 (95%CI 0.4 to 0.9) | |
| Objective cardiac testing at 30 days | 1 (Mahler et al., 2015 *)/282 | 56.7% | 68.8% p=0.04^†^ |
| Revascularisations at index visit (%)  Urgent post discharge revascularisations (%)  Planned post discharge revascularisations (%) | 1 (Sanchis et al., 2009 )/320 | 8.1%  1.3%  5% | 18.1% p=0.01^†^  2.5% p=0.07^†^  0.6% p=0.04^†^ |
| Discharge without objective cardiac testing | 1 Mahler et al., 2015 *)/282 | 39.7% | 18.4% p<0.00 |
| Use and implementation/application outcomes^‡^ | | | |
| Number (%) classified as low risk by the diagnostic pathway incorporating CPR but admitted to hospital | 1 (Than et al., 2014 )/542 | 35 (12.9%)§ |  |

HR – hazard ratio; OR – odds ratio; CPR – clinical prediction rule

*primary outcome; †p value for difference between intervention and control groups; ‡only relevant to the intervention arm of studies where use of the CPR was discretionary; §none received a diagnosis of acute coronary syndrome

Results of studies of diagnostic CPRS for suspected (bacterial) pneumonia by outcome

| Outcomes | Number of trials/N of trial or comparison | Result | |
| --- | --- | --- | --- |
|  |  | **Intervention** | **Control** |
| Patient outcomes | | | |
| Unfavourable clinical outcome | 2 (Ferrero et al., 2015 )/65  (Torres et al., 2014 )/120 | OR 0.7 (95%CI 0.1 to 3.4)  OR 1.0 (95%CI 0.2 to 3.6) | |
| Process outcomes | | | |
| Proportion of visits resulting in return to ED 2 weeks after initial visit | 1 (McGinn et al., 2013 [5]/984‡ | 0.7% | 0.5% p>0.9 |
| Proportion of visits resulting in outpatient clinic visits 2 weeks after initial visit | 1 (McGinn et al., 2013 [5]/984‡ | 7.7% | 11.3% p=0.1 |
| Clinical decisions/appropriateness of clinical decisions | | | |
| Antibiotic prescriptions | 3 (Ferrero et al., 2015 *)/65  (Torres et al., 2014 *)/120  (McGinn et al., 2013 *)/395 | OR 0.2 (95%CI 0.1 to 0.6)  OR 0.13 (95%CI 0.05 to 0.35)  RR 0.79 (95%CI 0.64 to 0.98)^†^ | |
| Chest radiographs ordered | 1(McGinn et al., 2013 )/395 | RR 0.98 (95%CI 0.60 to 1.62)^†^ | |
| Use and implementation/application outcomes | | | |
| Decision support tool opened | 1(McGinn et al., 2013 )/395 | 42.5% |  |
| CPR calculator opened | 1(McGinn et al., 2013 )/395 | 41.5% |  |

CPR – clinical prediction rule; OR – odds ratio; RR – risk ratio

*primary outcome; ^†^ adjusted for age; ‡This study evaluated two CPRs for different conditions. For this outcome, the data from both evaluations are combined.

Results of studies of diagnostic CPRs for suspected ankle or mid-foot fracture by outcome

| Outcomes | Number of trials/N of trial or comparison | Result | |
| --- | --- | --- | --- |
|  |  | **Intervention** | **Control** |
| Patient outcomes | | | |
| Patients satisfied with care (%) | 1 (Auleley et al., 1997 )/1911 | 96% | 98% |
| Patient satisfaction rating  (median score on 5 point scale) | 1 (Fan et al., 2006 )/124 | 4 | 4 p=0.34^†^ |
| Process outcomes | | | |
| Total length of stay in ED (minutes) | 1 (Fan et al., 2006 *)/124 | MD -6.7 (95%CI -20.9 to 7.4) | |
| Time from triage to registration  (mean minutes) |  | 8.0 | 8.0 p=0.80† |
| Time from registration to room assignment (mean minutes) |  | 20.0 | 13.0 p=0.05† |
| Time from room assignment to clinician assessment (mean minutes) |  | 25.0 | 19.0 p=0.16† |
| Time from clinician assessment to disposition (mean minutes) |  | 21.0 | 27.0 p=0.62 |
| Clinical decisions/appropriateness of decisions | | | |
| Radiography requests (%) | 2 (Auleley et al., 1997 *)/1911  (Fan et al., 2006 )/124 | 76%  94% | 99% p=0.03^†^  89% p=0.36^†^ |
| Number of fractures in those not x-rayed | 1 (Auleley et al., 1997 )/1911 | 3 | 0^‡^ |
| Use and implementation/application outcomes^§^ | | | |
| Use of the data collection for containing the CPR | 1 (Auleley et al.,1997 )/1911 | 75% |  |
| Requested x-ray when CPR recommended x-ray |  | 99% |  |
| Requested x-ray when CPR recommended no x-ray |  | 21% |  |

CPR – clinical prediction rule; MD – mean difference; ED – emergency department

*primary outcome; ^†^p value for difference between intervention and control groups; ‡There was no active follow-up of participants in this trial and individuals with persistent pain or difficulty walking may have presented for care elsewhere; §only relevant to the intervention arm of studies where use of the CPR was discretionary

Results of single studies of diagnostic CPRS for different clinical conditions

| Trial/N of trial | Outcomes | Result | |
| --- | --- | --- | --- |
|  |  | **Intervention** | **Control** |
| Children with fracture, dislocation or joint effusion of the extremities | | | |
| Klassen et al., 1993 /991 | Radiography requests*  CPR only  CPR as triage test  Missed fractures (%)  CPR only  CPR as triage test  Time spent in ED (mean hrs) | RR 0.94 (95%CI 0.89 to 0.99)  RR 1.12 (95%CI 1.08 to 1.16) | |
|  |  | 3.2%  0%  3.3 (SD 1.7) | 0%  0%  3.6 (SD 1.5) ( p<0.001)† |
| Suspicious pigmented lesions | | | |
| Walter et al., 2012 /1580 | Appropriate referral rate*^‡^  Benign lesions appropriately managed in PC (%)  Sensitivity  Specificity  Lesions referred (%) | % difference -8.1 (95%CI -18.0 to 1.8)  % difference 0.5 (95%CI -0.6 to 2.0) | |
|  |  | 98.5%  84.4%  29.8% | 95.7% (=0.26)†  90.6% (p<0.001)†  22.4% (p=0.001)† |
| Pulmonary embolism | | | |
| Rodger et al., 2006 /398 | Venous thromboembolic event rate during 3 month f-up*  Total bleeding episodes during 3 months follow-up  Diagnostic imaging tests performed (mean no./patient) | % difference -0.6 (95%CI -4.1 to 2.9) | |
|  |  | 5  1.36 | 2 (p=0.45)†  1.9 (p<0.001)† |
| Gastro-oesophageal reflux disease | | | |
| Horowitz et al., 2007 /132 | Relief of symptoms*  Improvement in daily activities  Management costs (US$)  Number of GP visits  Number of specialist referrals  Cost of medications (US$)  Number of imaging diagnostic tests | MD 2.5 (95%CI 1.49 to 3.51)  MD 0.6 (95%CI 0.18 to 1.02)  MD -138.00 (95%CI -230.70 to -45.30)  MD -0.3 (95%CI -0.59 to -0.00)  MD -0.36 (95%CI -0.65 to -0.09)  MD -68.00 (95%CI -269.40 to 133.38)  MD -0.10 (95%CI -0.23 to 0.03) | |
| Complete acute bowel obstruction | | | |
| Bogusevicius et al., 2002 /80 | Sensitivity*  Specificity*  Time to make diagnosis (hrs)  Mortality | 100% (95%CI 86.2 to 1)  87.5% (95%CI 6.39 to 96.5)  1  5.0% | 100% (95%CI 87.5 to 1)  76.9% (95%CI 49.7 to 91.8)  16 (p<0.001)§  0.0% |
| Clinically important brain injury | | | |
| Stiell et al., 2010 /4531 | % change in CT scan rates from before to after*  Missed brain injuries during the ED visit  % of clinicians using rule  % of clinicians accurately completing rule | 13.3% (95%CI 9.7 to 17.0)  0  78%  82.5% | 6.7% (95%CI 2.6 to 10.8) (p=0.16)§  0 |
| Cervical spine fracture | | | |
| Stiell et al., 2009 /11,824 | % change in cervical spine imaging before to after*  Missed cervical spine fractures during the ED visit  % of clinicians using rule  % of clinicians accurately completing rule | -12.8 (95%CI -9.2 to -16.3)  0  85.7%  82.9% | 12.5 (95%CI 7.2 to 18.2)(p=0.00)^§^  0 |

CPR – clinical prediction rule; SD – standard deviation; MD – mean difference; OR – odds ratio; RR – risk ratio

*primary outcome; †p value for difference between intervention and control group; ^‡^ no. of referred lesions secondary care experts decided to biopsy or monitor/number referred; § p value for relative change in mean imaging rates from the before period to the after period between intervention and control hospitals.

References for data presented in Additional Files

1. Little P, Hobbs FD, Moore M, Mant D, Williamson I, McNulty C, Cheng YE, Leydon G, McManus R, Kelly J, et al: **Clinical score and rapid antigen detection test to guide antibiotic use for sore throats: randomised controlled trial of PRISM (primary care streptococcal management).** *BMJ* 2013, **347:**f5806.

2. McIsaac WJ, Goel V, To T, Permaul JA, Low DE: **Effect on antibiotic prescribing of repeated clinical prompts to use a sore throat score: lessons from a failed community intervention study.** *J Fam Pract* 2002, **51:**339-344.

3. Worrall G, Hutchinson J, Sherman G, Griffiths J: **Diagnosing streptococcal sore throat in adults: randomized controlled trial of in-office aids.** *Can Fam Physician* 2007, **53:**666-671.

4. McIsaac WJ, Goel V: **Effect of an explicit decision-support tool on decisions to prescribe antibiotics for sore throat.** *Med Decis Making* 1998, **18:**220-228.

5. McGinn TG, McCullagh L, Kannry J, Knaus M, Sofianou A, Wisnivesky JP, Mann DM: **Efficacy of an evidence-based clinical decision support in primary care practices: a randomized clinical trial.** *JAMA Intern Med* 2013, **173:**1584-1591.

6. Wellwood J, Johannessen S, Spiegelhalter DJ: **How does computer-aided diagnosis improve the management of acute abdominal pain?** *Ann R Coll Surg Engl* 1992, **74:**40-46.

7. Liu JL, Wyatt JC, Deeks JJ, Clamp S, Keen J, Verde P, Ohmann C, Wellwood J, Dawes M, Altman DG: **Systematic reviews of clinical decision tools for acute abdominal pain.** *Health Technol Assess* 2006, **10:**1-167, iii-iv.

8. Douglas CD, Macpherson NE, Davidson PM, Gani JS: **Randomised controlled trial of ultrasonography in diagnosis of acute appendicitis, incorporating the Alvarado score.** *BMJ* 2000, **321:**919-922.

9. Farahnak M, Talaei-Khoei M, Gorouhi F, Jalali A, Gorouhi F: **The Alvarado score and antibiotics therapy as a corporate protocol versus conventional clinical management: randomized controlled pilot study of approach to acute appendicitis.** *Am J Emerg Med* 2007, **25:**850-852.

10. Lintula H, Kokki H, Kettunen R, Eskelinen M: **Appendicitis score for children with suspected appendicitis. A randomized clinical trial.** *Langenbecks Arch Surg* 2009, **394:**999-1004.

11. Lintula H, Kokki H, Pulkkinen J, Kettunen R, Grohn O, Eskelinen M: **Diagnostic score in acute appendicitis. Validation of a diagnostic score (Lintula score) for adults with suspected appendicitis.** *Langenbecks Arch Surg* 2010, **395:**495-500.

12. Roukema J, Steyerberg EW, van der Lei J, Moll HA: **Randomized trial of a clinical decision support system: impact on the management of children with fever without apparent source.** *J Am Med Inform Assoc* 2008, **15:**107-113.

13. de Vos-Kerkhof E, Nijman RG, Vergouwe Y, Polinder S, Steyerberg EW, van der Lei J, Moll HA, Oostenbrink R: **Impact of a clinical decision model for febrile children at risk for serious bacterial infections at the emergency department: a randomized controlled trial.** *PLoS One* 2015, **10:**e0127620.

14. Lacroix L, Manzano S, Vandertuin L, Hugon F, Galetto-Lacour A, Gervaix A: **Impact of the lab-score on antibiotic prescription rate in children with fever without source: a randomized controlled trial.** *PLoS One* 2014, **9:**e115061.

15. Sanchis J, Bosch X, Bodi V, Nunez J, Doltra A, Heras M, Mainar L, Santas E, Bragulat E, Garcia-Alvarez A, et al: **Randomized comparison between clinical evaluation plus N-terminal pro-B-type natriuretic peptide versus exercise testing for decision making in acute chest pain of uncertain origin.** *Am Heart J* 2010, **159:**176-182.

16. Than M, Aldous S, Lord SJ, Goodacre S, Frampton CM, Troughton R, George P, Florkowski CM, Ardagh M, Smyth D, et al: **A 2-hour diagnostic protocol for possible cardiac chest pain in the emergency department: a randomized clinical trial.** *JAMA Intern Med* 2014, **174:**51-58.

17. Mahler SA, Riley RF, Hiestand BC, Russell GB, Hoekstra JW, Lefebvre CW, Nicks BA, Cline DM, Askew KL, Elliott SB, et al: **The HEART Pathway randomized trial: identifying emergency department patients with acute chest pain for early discharge.** *Circ Cardiovasc Qual Outcomes* 2015, **8:**195-203.

18. Ferrero F, Adrian Torres F, Dominguez P, Ossorio MF: **Efficacy and safety of a decision rule for using antibiotics in children with pneumonia and vaccinated against pneumococcus. A randomized controlled trial.** *Arch Argent Pediatr* 2015, **113:**397-403.

19. Torres FA, Pasarelli I, Cutri A, Ossorio MF, Ferrero F: **Impact assessment of a decision rule for using antibiotics in pneumonia: a randomized trial.** *Pediatr Pulmonol* 2014, **49:**701-706.

20. Auleley GR, Ravaud P, Giraudeau B, Kerboull L, Nizard R, Massin P, Garreau de Loubresse C, Vallee C, Durieux P: **Implementation of the Ottawa ankle rules in France. A multicenter randomized controlled trial.** *JAMA* 1997, **277:**1935-1939.

21. Fan J, Woolfrey K: **The effect of triage-applied Ottawa Ankle Rules on the length of stay in a Canadian urgent care department: a randomized controlled trial.** *Acad Emerg Med* 2006, **13:**153-157.

22. Klassen TP, Ropp LJ, Sutcliffe T, Blouin R, Dulberg C, Raman S, Li MM: **A randomized, controlled trial of radiograph ordering for extremity trauma in a pediatric emergency department.** *Ann Emerg Med* 1993, **22:**1524-1529.

23. Walter FM, Morris HC, Humphrys E, Hall PN, Prevost AT, Burrows N, Bradshaw L, Wilson EC, Norris P, Walls J, et al: **Effect of adding a diagnostic aid to best practice to manage suspicious pigmented lesions in primary care: randomised controlled trial.** *BMJ* 2012, **345:**e4110.

24. Rodger MA, Bredeson CN, Jones G, Rasuli P, Raymond F, Clement AM, Karovitch A, Brunette H, Makropoulos D, Reardon M, et al: **The bedside investigation of pulmonary embolism diagnosis study: a double-blind randomized controlled trial comparing combinations of 3 bedside tests vs ventilation-perfusion scan for the initial investigation of suspected pulmonary embolism.** *Arch Intern Med* 2006, **166:**181-187.

25. Horowitz N, Moshkowitz M, Leshno M, Ribak J, Birkenfeld S, Kenet G, Halpern Z: **Clinical trial: evaluation of a clinical decision-support model for upper abdominal complaints in primary-care practice.** *Aliment Pharmacol Ther* 2007, **26:**1277-1283.

26. Bogusevicius A, Maleckas A, Pundzius J, Skaudickas D: **Prospective randomised trial of computer-aided diagnosis and contrast radiography in acute small bowel obstruction.** *Eur J Surg* 2002, **168:**78-83.

27. Stiell IG, Clement CM, Grimshaw JM, Brison RJ, Rowe BH, Lee JS, Shah A, Brehaut J, Holroyd BR, Schull MJ, et al: **A prospective cluster-randomized trial to implement the Canadian CT Head Rule in emergency departments.** *CMAJ* 2010, **182:**1527-1532.

28. Stiell IG, Clement CM, Grimshaw J, Brison RJ, Rowe BH, Schull MJ, Lee JS, Brehaut J, McKnight RD, Eisenhauer MA, et al: **Implementation of the Canadian C-Spine Rule: prospective 12 centre cluster randomised trial.** *BMJ* 2009, **339:**b4146.
